# *Tobacco mosaic virus* movement protein complements a *Potato spindle tuber viroid* RNA mutant impaired for mesophyll entry but not mutants unable to enter the phloem

**DOI:** 10.1371/journal.ppat.1011062

**Published:** 2022-12-27

**Authors:** Jian Wu, David M. Bisaro

**Affiliations:** 1 Department of Molecular Genetics, Center for Applied Plant Sciences, Center for RNA Biology, and Infectious Diseases Institute, The Ohio State University, Columbus, Ohio, United States of America; 2 State Key Laboratory for Managing Biotic and Chemical Threats to the Quality and Safety of Agro-products, Institute of Plant Virology, Ningbo University, Ningbo, China; Agriculture and Agri-Food Canada, CANADA

## Abstract

*Tobacco mosaic virus* movement protein (TMV MP) is essential for virus spread between cells. To accomplish its task, TMV MP binds viral RNA, interacts with components of the cytoskeleton, and increases the size exclusion limit (SEL) of plasmodesmata. Plasmodesmata are gated intercellular channels that allow passage of small molecules and macromolecules, including RNA and protein, between plant cells. Moreover, plasmodesmata are diverse and those connecting different cell types appear to have unique mechanisms to regulate macromolecular trafficking, which likely contributes to the establishment of distinct cell boundaries. Consequently, TMV MP might be competent to mediate RNA transport through some but not all plasmodesmal gates. Due to a lack of viral mutants defective for movement between specific cell types, the ability of TMV MP in this regard is incompletely understood. In contrast, a number of trafficking impaired *Potato spindle tuber viroid* (PSTVd) mutants have been identified. PSTVd is a systemically infectious non-coding RNA that nevertheless can perform all functions required for replication as well as cell-to-cell and systemic spread. Previous studies have shown that PSTVd employs different structure and sequence elements to move between diverse cell types in host plants, and mutants defective for transport between specific cell types have been identified. Therefore, PSTVd may serve as a tool to analyze the functions of MPs of viral and cellular origin. To probe the RNA transport activity of TMV MP, transgenic plants expressing the protein were inoculated with PSTVd mutants. Remarkably, TMV MP complemented a PSTVd mutant defective for mesophyll entry but could not support two mutants impaired for phloem entry, suggesting it fails to productively interface with plasmodesmata at the phloem boundary and that additional viral and host factors may be required. Consistent with this idea, TMV co-infection, but not the combination of MP and coat protein (CP) expression, was able to complement one of the phloem entry mutants. These observations suggest that phloem loading is a critical impediment to establishing systemic infection that could involve the entire ensemble of TMV proteins. They also demonstrate a novel strategy for analysis of MPs.

## Author summary

TMV 30 kDa movement protein (TMV MP) mediates virus spread in part by increasing the size exclusion limit of plasmodesmata, intercellular channels that provide cytoplasmic continuity between plant cells and regulate trafficking of protein and RNA. However, plasmodesmata connecting different cell types have unique gating mechanisms that likely contribute to the establishment of distinct cell boundaries. Thus, TMV MP may enable viral RNA passage through some but perhaps not all plasmodesmal gates. In particular, its ability to support RNA trafficking between bundle sheath cells and phloem parenchyma, a key step in phloem loading, is unknown. To address this question, available PSTVd RNA mutants defective for trafficking between specific cell types were used to infect transgenic *Nicotiana benthamiana* plants expressing TMV MP. TMV MP complemented a PSTVd mutant defective for invasion of underlying mesophyll following inoculation to epidermal cells. However, it failed to complement two mutants that are unable to enter the phloem, although one could be complemented by co-infection with TMV. These observations suggest that TMV MP alone does not productively interact with plasmodesmata at the phloem boundary and that additional viral and host factors are required. They further confirm that phloem loading is more complex than other types of cell-to-cell movement, and demonstrate a novel approach for the analysis of MPs.

## Introduction

Intercellular transport of macromolecules is crucial for all multicellular eukaryotes, yet our understanding of the principles and mechanisms underlying this process is far from complete and new analytical approaches are needed. *Potato spindle tuber viroid* (PSTVd) and *Tobacco mosaic virus* (TMV) continue to be important models for the analysis of RNA trafficking in plants. TMV has a plus-sense single-stranded RNA genome of ~6.5 kb that specifies four proteins. Conversely, PSTVd is a circular non-coding RNA of 359 nucleotides, thus all functions required for host plant infection are mediated by sequence and structure elements within the genomic RNA [1–3]. Viroids and viruses accomplish systemic infection by two distinct mechanisms; cell-to-cell spread and long-distance transport through the phloem. Spread between cells occurs through plasmodesmata, gated channels that provide cytoplasmic continuity and regulate the transport of macromolecules, including RNA and protein [4–6]. While viroid RNAs, with the support of host factors, can traverse plasmodesmata and traffick through the phloem transport system, viruses additionally require specialized movement proteins [7,8]. The first characterized movement protein, TMV 30 kDa protein (TMV MP), enables viral movement between cells by directly binding viral RNA, interacting with components of the cytoskeleton as well as the ER and associated actin network, and increasing the size exclusion limit (SEL) of plasmodesmata [9–14]. (SEL is defined by the size of the largest molecules that can pass through plasmodesmata). However, plasmodesmata connecting different cell types have unique structures and gating mechanisms. For instance, different SELs and structures have been observed in particular cell types, such as cells of the Arabidopsis root tip, and specialized plasmodesmata connecting phloem companion cells and sieve elements [15–17]. Further, as we have learned from analysis of PSTVd mutants, plasmodesmata between different cell types (including upper epidermis, palisade mesophyll, spongy mesophyll, bundle sheath, and phloem parenchyma) recognize different RNA structure or sequence elements, and requirements for viroid RNA trafficking are often unique and directional [18–22].

Mechanisms of TMV RNA spread between cells, and phloem loading and unloading, are incompletely understood and may involve other viral factors in addition to MP, such as coat protein (CP), replicase, or replication complexes [23–25]. Previous studies using MP-expressing transgenic tobacco plants showed that MP can mediate spread of macromolecules such as FITC-dextran between mesophyll and bundle sheath cells [10], but not between bundle sheath and phloem parenchyma [26]. Further, CP is essential for phloem-mediated accumulation of TMV [27,28], suggesting that phloem transport involves encapsidation of viral RNA into virions or a viral RNA-CP complex. However, CP is not required for TMV invasion of phloem parenchyma in inoculated leaves [29], although CP interaction with specific host factors could influence the efficiency of systemic spread [30]. Interestingly, results of a recent study indicate that negative regulation of salicylic acid signaling by CP also promotes long systemic spread [31], suggesting a role independent of its interaction with viral RNA. Additionally, studies have implicated the TMV 123/183 kDa replicase proteins in phloem loading, and these may also participate indirectly by altering the expression of auxin-responsive genes [32]. In any case, plant viruses in general can move between individual epidermal and mesophyll cells within hours while phloem loading takes considerably longer, suggesting the involvement of different host factors and/or a unique structure of plasmodesmata connecting bundle sheath cells and phloem [33]. Therefore, it is reasonable to hypothesize that TMV MP might vary in its ability to support cell-to-cell spread vs. phloem loading of viral RNA. Because genetic studies to address this question require RNAs defective for trafficking between specific cell types, we speculated that PSTVd mutants might prove useful. Further, because TMV MP can increase plasmodesmal SEL and transit itself to adjacent cells in the absence of other viral proteins [10,34], we examined PSTVd trafficking in transgenic plants expressing native TMV MP.

In this study, we show that TMV MP complements a PSTVd mutant that is unable to enter the mesophyll following rub-inoculation of epidermal cells, but cannot complement two mutants that are unable to enter the phloem. In addition, transient expression of CP in MP-expressing plants also failed to enable phloem loading of these mutants, although one was complemented by co-infection with TMV. These results suggest that TMV MP fails to productively interface with plasmodesmata at the phloem boundary, and that additional viral and host factors may be involved in phloem loading. These findings are consistent with the idea that gating systems for phloem entry are more complex than those at other boundaries. They also highlight the potential of PSTVd mutants as genetic tools to study the abilities of viral and endogenous MPs to traffick RNA between specific cell types.

## Results and discussion

### PSTVd mutants and transgenic plants expressing TMV MP

**Fig 1:**
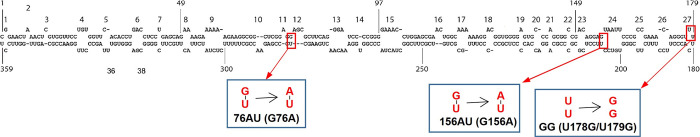
Locations of the PSTVd GG, 76AU, and 156AU mutants. PSTVd mutations U178G/U179G (GG), G76A (76AU), and G156A (156AU) are highlighted on the secondary structure of PSTVd. Loops/bulges in the circular 359 nt genomic RNA are numbered 1 through 27.

Three previously characterized PSTVd mutants were selected to test the ability of TMV MP to mediate RNA movement between different cell types: U178G/U179G, G76A, and G156A (hereafter GG, 76AU, and 156AU, respectively) [21,22]. The secondary structure of PSTVd contains 27 loops or bulges and 26 base-paired stems (Fig 1). GG disrupts the 3D structure of loop 27, whereas 76AU and 156AU convert essential G-U pairs on two distinct stem regions to A-U. PSTVd GG is unable to enter palisade mesophyll, the layer of cells that directly underlies the upper epidermis, when rub-inoculated to epidermal cells on the upper leaf surface. However, it can spread between all cell types, including from palisade mesophyll cells to upper epidermis, when inoculated by phloem injection [21]. Mutants 76AU and 156AU are defective for phloem entry from bundle sheath cells following rub-inoculation but can exit the phloem and move between all cell types if directly injected into phloem [22]. The PSTVd intermediate strain in which the mutants were constructed infects *Nicotiana benthamiana* (Nb) but not *N*. *tabacum*, while TMV infects both species. Nb plants expressing native TMV MP (Nb+MP) under control of the constitutive *Cauliflower mosaic virus* 35S promoter were reported in a previous study and shown to complement a TMV mutant (TE1) defective for cell-to-cell movement [35]. The Nb+MP plants do not have a visually obvious phenotype, suggesting that MP does not randomly mobilize RNAs. The presence of MP-specific transcripts in the transgenic Nb+MP plants was confirmed by RT-PCR in 9 randomly selected plants (S1 Fig).

Functional MP expression in Nb+MP plants was confirmed by monitoring systemic spread of trafficking-defective mutant TMV-GFP(TE1), which we constructed by introducing the original TE1 frameshift mutation into a TMV-GFP vector [36,37]. This involved deletion of a single nucleotide (A4931) in the MP coding region, resulting in a truncated protein of 16 amino acids [35]. Six Nb and Nb+MP plants were co-inoculated by agroinfiltration with pCB301 vectors to express the TMV-GFP(TE1) mutant and GUS (control), and as a positive control another six Nb plants were similarly co-inoculated with TMV and TMV-GFP(TE1). At nine days post-inoculation, GFP fluorescence and MP expression were detected in systemic leaves of Nb+MP plants co-inoculated with TMV-GFP(TE1) and GUS, and Nb plants co-inoculated with TMV and TMV-GFP(TE1) (S2 Fig). Because MP expression could occur from the transgene, wild type virus, or mutant virus, presence of the mutant TE1 MP sequence was confirmed by sequencing six mutant progeny from each of these two groups of plants. In contrast, in Nb plants co-inoculated with pCB301 vectors to express TMV-GFP(TE1) and GUS, GFP fluorescence was confined to agroinfiltration sites on lower leaves and was never observed in systemic leaves, indicating that GUS expression was incapable of complementing TMV-GFP(TE1). Thus, transgenic MP expression is sufficient to complement the trafficking-defective TMV-GFP(TE1) mutant.

### TMV MP complements a PSTVd mutant deficient for mesophyll entry

**Fig 2:**
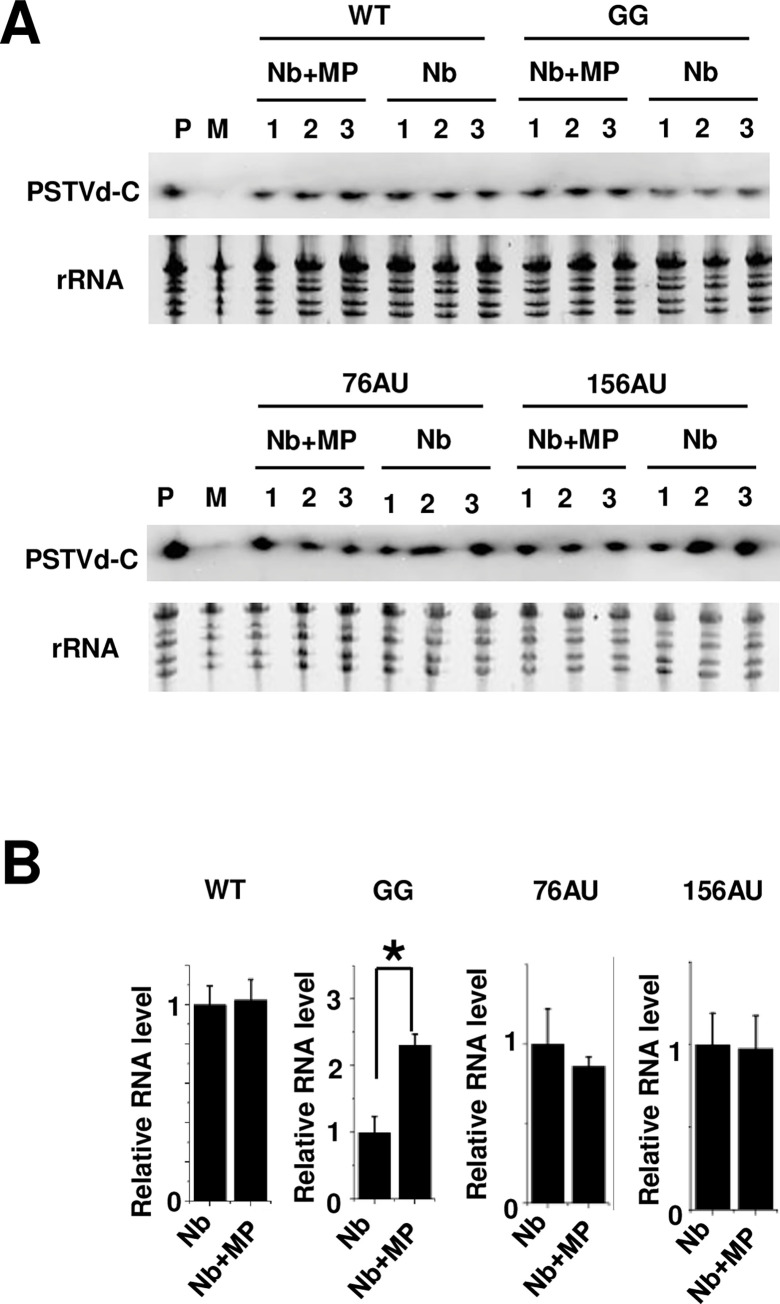
TMV MP increases accumulation of the GG mutant, but not 76AU and 156AU mutants, in inoculated leaves. (A) PSTVd accumulation in rub-inoculated leaves. *In vitro* transcripts of WT PSTVd and GG, 76AU, and 156AU mutants were rub-inoculated onto the upper surfaces of the first two true leaves of Nb and Nb+MP plants. Inoculated leaves were collected at 10 dpi and pooled. Total RNA samples were analyzed by RNA blot to detect PSTVd RNA (circular form, PSTVd-C), and levels were normalized to ribosomal RNA (rRNA) loading controls. Three biological replicates are indicated by number, and each included four plants. P, positive control, M, Mock. (B) RNA blot signals from (A) were quantified using Quantity One software. For each treatment the Nb+MP group was normalized to the Nb group, which was set to 1. Shown are mean +/- SD values of the three biological replicates. Asterisk indicates significant difference (p < 0.05) by Student’s *t* test.

The Nb+MP plants and PSTVd mutants described above were used to assess the ability of TMV MP to mediate RNA trafficking between diverse cell types. Full-length *in vitro* transcripts of wild type (WT) PSTVd and each of the mutants were rub-inoculated onto the upper surfaces of the first two true leaves of Nb and Nb+MP plants. Accumulation of PSTVd RNAs in the inoculated leaves was analyzed in three biological replicates with 4 plants each. Extracts were obtained 10 days post-inoculation (dpi) and levels of circular PSTVd RNA, the functional form of the genome, were determined by RNA blot (Fig 2A). When RNA levels were quantified and compared between Nb and Nb+MP plants, no significant differences in PSTVd accumulation were observed in inoculated leaves following WT, 76AU, and 156AU infection (Fig 2B), indicating that TMV MP does not generally enhance PSTVd replication or spread. However, PSTVd RNA levels were significantly increased in Nb+MP plants compared to Nb plants after GG infection (Fig 2B), suggesting TMV MP may enable mesophyll entry of the GG mutant, thereby increasing the number of infected cells.

**Fig 3:**
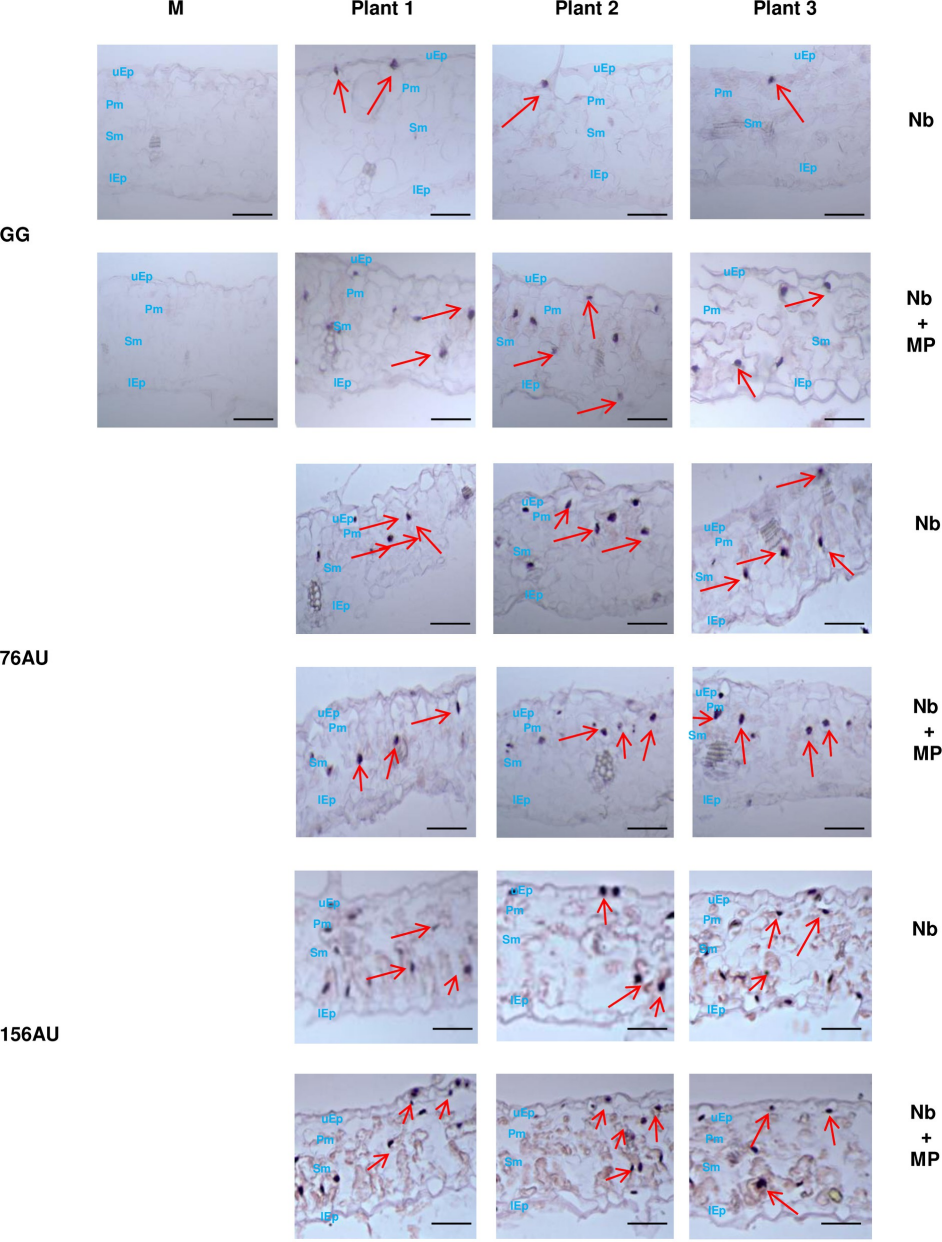
TMV MP enables mesophyll entry of the GG mutant but does not affect the cellular distribution of 76AU and 156AU mutants in inoculated leaves. Transverse section *in situ* hybridization was conducted to analyze the accumulation of the GG, 76AU, and 156AU mutants in different cell types. Rub-inoculated leaves from three Nb and three Nb+MP plants infected with GG, 76AU, or 156AU were collected at 10 dpi. Images presented are representative of > 40 visual fields each. Mock negative controls (M) are also shown. Purple dots, some highlighted by red arrows, are viroid hybridization signals in nuclei. Bars = 12 μm. uEp, upper epidermis; Pm, palisade mesophyll; Sm, spongy mesophyll; lEp, lower epidermis.

To test this hypothesis, *in situ* hybridization was performed on transverse sections of leaves from Nb and Nb+MP plants that were rub-inoculated with the GG mutant. Confirming previous results [21], PSTVd GG was observed only in upper epidermal (uEp) cells of Nb plants (Fig 3). In contrast, the GG mutant was detected in all cell types, including uEp, palisade mesophyll (Pm), spongy mesophyll (Sm), and lower epidermis (lEp), in similarly inoculated leaves of Nb+MP plants (Fig 3), allowing us to conclude that TMV MP enables passage of the GG mutant through plasmodesmata connecting upper epidermis and palisade mesophyll. Transverse section *in situ* hybridization was also performed on plants that were rub-inoculated with the 76AU and 156AU mutants. Unlike the GG mutant, no obvious differences were observed in the cellular distribution of these PSTVd mutants in inoculated leaves of Nb compared to Nb+MP plants (Fig 3).

### TMV MP does not complement PSTVd mutants deficient for phloem entry

To test whether TMV MP can mediate PSTVd trafficking from bundle sheath cells to phloem, the ability of WT, GG, 76AU, and 156AU to systemically infect plants was assessed at 10-, 20-, and 30 dpi following rub inoculation of eight Nb and Nb+MP plants. As local infection is prerequisite for systemic infection, the presence of circular PSTVd RNA in rub-inoculated leaves was confirmed at 10 dpi in all cases (8/8 Nb and Nb+MP plants) (S3A Fig). As observed previously (Fig 2B), in inoculated leaves increased PSTVd RNA accumulation was observed only in Nb+MP plants compared to Nb plants after GG infection, and not following infection with WT, 76AU, or 156AU (S3B Fig). At this 10 day time point, PSTVd WT and mutants had not yet reached upper systemic leaves (S3C Fig).

**Fig 4:**
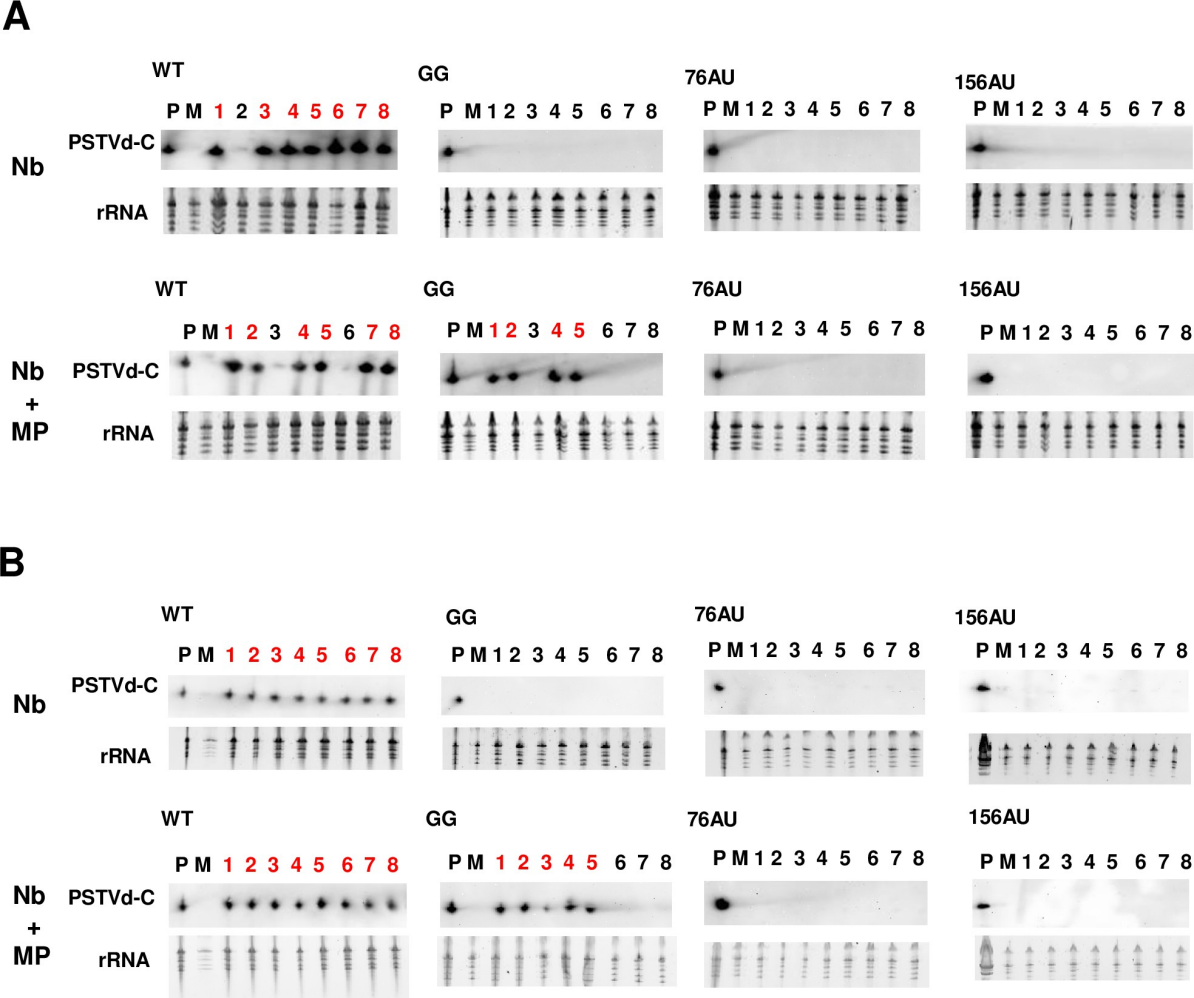
TMV MP enables systemic infection by the GG mutant, but not 76AU and 156AU mutants. Systemic infection at 20 and 30 dpi was monitored by RNA blot. *In vitro* transcripts representing WT PSTVd and the GG, 76AU, and 156AU mutants were rub-inoculated onto leaves of 2-week-old Nb and Nb+MP plants, with 8 plants in each group. Systemic (upper) leaves were collected at (A) 20 dpi and (B) 30 dpi. RNA was isolated and RNA blots performed to detect circular PSTVd RNA. P, positive control, M, mock. Numbers indicate individual plants, with red indicating positive PSTVd signal. Loading control was rRNA.

By 20 dpi, WT PSTVd systemically infected 7/8 Nb plants and 6/8 Nb+MP plants. Although GG failed to systemically infect Nb plants, 4/8 Nb+MP plants were infected, consistent with our previous observation that this mutant is capable of systemic infection if the upper epidermis-palisade mesophyll boundary is bypassed [21] (Fig 4A). In contrast, 76AU and 156AU mutants were unable to systemically infect Nb or Nb+MP plants at 20 dpi. By 30 dpi, WT PSTVd systemically infected all 8 Nb and Nb+MP plants, while 5/8 Nb+MP plants were infected by the GG mutant. No systemic infection was detected in 76AU and 156AU inoculated plants even after 30 days (Fig 4B), suggesting that TMV MP is unable to complement mutants deficient for phloem loading, and specifically for trafficking from bundle sheath cells to phloem [22].

### Systemic infection of Nb+MP plants by the GG mutant is not due to reversion

We and others have shown that PSTVd replication occurs with a high error rate, generating large numbers of variants in progeny populations that can be considered quasispecies [22,38,39]. Thus, it is possible that apparent complementation leading to systemic infectivity of the GG mutant (178G/179G) observed in Nb+MP plants might be the result of reversion to WT and/or functional single mutants (U178G or U179G) [21]. It is also conceivable that additional beneficial mutations could compensate the original mutations and enable systemic infection. To rule out these possible scenarios, full-length progeny genomes from both inoculated (local, L) and systemic (S) leaves of three GG-infected Nb and Nb+MP plants were analyzed by Sanger sequencing.

As expected, in Nb and Nb + MP plants infected with WT PSTVd, all progeny genomes recovered from local and systemic leaves were WT (Table 1). Since the GG mutant is defective for mesophyll entry in Nb plants systemic infection is impossible, and only genomes from local leaves of Nb plants could be sequenced. Four of the six progeny genomes sequenced maintained the double mutation, and partial reversion (U178G/U179G to U179G) was detected in the remaining two cases. Similar partial reversion was observed in our previous study [21]. In GG-infected Nb+MP plants, six progeny genomes from local leaves and five from systemic leaves were sequenced. Except for a single additional mutation (U283A) detected in one genome from local leaves and one partial reversion (U178G/U179G to U179G) observed in a genome from systemic leaves, all others retained the double mutation (Table 1). In total, the 178G/179G double mutation was maintained in 13 of 17 sequenced genomes, while one acquired a single additional mutation and 3 experienced partial reversion to U179G. Therefore, successful mesophyll entry of the GG mutant in Nb+MP plants, and ensuing systemic infection, is unlikely to be caused by reversion and/or additional mutations, confirming TMV MP mediated complementation.

**Table 1:** Progeny sequences from inoculated and systemic leaves of Nb and Nb+MP plants infected with WT PSTVd or the GG mutant (U178G/U179G).

	Nb			Nb+MP		
	Plant 1	Plant 2	Plant 3	Plant 1	Plant 2	Plant 3
**WT-L**	WT(2)	WT(2)	WT(2)	WT(2)	WT(2)	WT(3)
**WT-S**	WT(2)	WT(3)	WT(3)	WT(3)	WT(3)	WT(2)
**GG-L**	U178G/U179G(2)	U178G/U179G(1) U179G(1)	U178G/U179G(1)U179G(1)	U178G/U179G(2)	U178G/U179G(2)	U178G/U179G(1)U178G/U179G/U283A(1)
**GG-S**	NA	NA	NA	U178G/U179G(1)U179G(1)	U178G/U179G(1)	U178G/U179G(2)

Progeny obtained from both inoculated (L, local) or systemic (S) leaves of three Nb and three Nb+MP plants inoculated with WT PSTVd or the GG mutant were cloned and sequenced. Numbers in parentheses indicate number of clones containing the sequence. NA, not applicable.

### TMV MP binds wild type and mutant PSTVd RNAs

**Fig 5:**
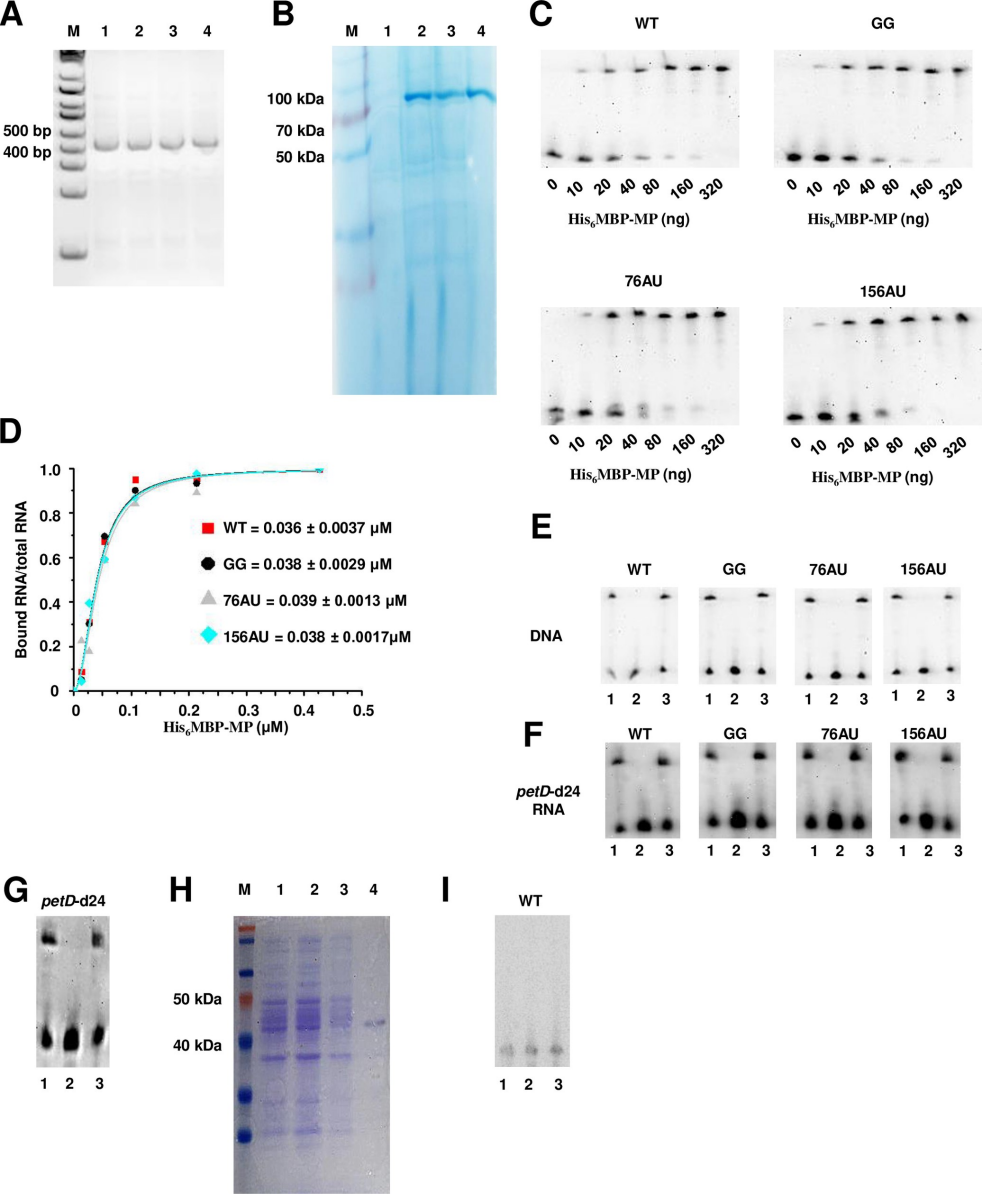
TMV MP binds WT PSTVd and GG, 76AU, and 156AU mutants. (A) Folded full-length PSTVd *in vitro* transcripts prepared for EMSA. After folding, PSTVd WT and mutant RNAs were tested for homogeneity by electrophoresis through 6% native polyacrylamide gel. The image was taken after ethidium bromide staining. Lanes 1–4 contained WT PSTVd, GG, 76AU, and 156AU, respectively. M, size marker. (B) Preparation of His_6_MBP-MP fusion protein (~73 kDa). M, size marker; lane 1, total protein extracted from *E*. *coli* strain BL21 prior to IPTG induction; lane 2, total protein following 0.1 M IPTG induction; lane 3, supernatant containing excess His_6_MBP-MP following metal affinity chromatography with Ni-NTA agarose and centrifugation; lane 4, His_6_MBP-MP eluted from the Ni-NTA agarose pellet fraction. Proteins were visualized by Coomassie blue staining following electrophoresis through 10% SDS-polyacrylamide gel. (C) EMSA assay. Folded and unlabeled PSTVd WT and mutant *in vitro* transcripts (1 ng) were incubated with increasing amounts of His_6_MBP-MP. Binding was analyzed by EMSA through 6% native polyacrylamide gel, followed by RNA blot using labeled PSTVd RNA probe. Three biological replicates were performed, and a representative replicate for each treatment is shown. (D) Binding was quantified by analyzing data from (C) using Quantity One software. Binding curves were plotted and fit using the Hill function of OriginPro 8.5 Software. Calculated protein concentrations at which half the RNA was bound are presented as mean +/-SD values of three replicates. (E) Competition assay. Folded and unlabeled PSTVd *in vitro* transcripts (1 ng) were incubated with 40 ng His_6_MBP-MP alone (lane 1), or in the presence of 100 ng ssDNA (lane 2) or dsDNA (lane 3), followed by 6% native polyacrylamide gel electrophoresis and RNA blotting to detect PSTVd RNA. (F) Competition assay was performed as described for panel E except single-stranded *petD*-d24 (ss*petD*-d24) RNA (sense) and double-stranded *petD*-d24 (ds*petD*-d24 RNA) instead of ssDNA and dsDNA were used. (G) Competition assay was performed as described for panel E except spinach *pet*D-d24 *in vitro* transcripts (positive control), instead of PSTVd *in vitro* transcripts, were used. (H) His_6_MBP (~43 kDa, negative control) was obtained by the same procedure used to prepare His_6_MBP-MP fusion protein, as described for panel B. (I) Competition assay performed as described in panel E, except 1 ng WT PSTVd and 40 ng His_6_MBP were used. His_6_MBP does not bind PSTVd RNA. (Note: experiments shown in panels G and I were performed together under the same conditions).

TMV MP binds viral RNA to enable spread [11,40]. To determine if impaired binding might be responsible for the inability of MP to complement PSTVd 76AU and 156AU, binding was directly tested by electrophoretic mobility shift assay (EMSA). Since PSTVd RNA in solution adopts a structure similar to that observed in plant cells [41], *in vitro* transcripts were prepared and folded to ensure the formation of homogenous structures. Ethidium bromide staining revealed a single band after 6% native polyacrylamide gel electrophoresis of *in vitro* transcripts of WT PSTVd and the GG, 76AU, and 156AU mutants, suggesting the formation of homogenous structures in all cases (Fig 5A). TMV MP was expressed as a six histidine, maltose binding protein fusion (His_6_MBP-MP) in *E*. *coli* strain BL21 and purified from cell extracts using metal affinity chromatography with Ni-NTA agarose (Fig 5B). MBP was chosen because it often enhances solubility of recombinant proteins and does not bind nucleic acids [42]. To perform EMSA, increasing amounts of protein (0 to 320 ng) were incubated with 1 ng PSTVd *in vitro* transcripts in 10 μl binding buffer. Following 6% native gel electrophoresis, RNA-protein complexes were visualized by RNA blot with labeled PSTVd probe. EMSA analysis showed that His_6_MBP-MP was able to bind WT PSTVd and all three mutants (Fig 5C). Binding curves were plotted using quantified EMSA data, and no significant differences were observed in relative binding efficiency of His_6_MBP-MP with WT PSTVd and the mutant RNAs. Under the conditions employed, half the RNA was bound at protein concentrations of 0.036 μM (WT), 0.038 μM (GG), 0.039 μM (76AU), and 0.038 μM (156AU) (Fig 5D). This is consistent with a previous study which showed that TMV MP binds with similar affinity to RNA and single-stranded DNA (ssDNA) through binding sites comprising only 4 to 7 nucleotides, but does not bind double-stranded DNA (dsDNA) [11]. Binding specificity of our fusion protein was confirmed by testing 1 ng folded PSTVd RNA transcripts with 40 ng His_6_MBP-MP alone, or in the presence of 100 ng heterologous ssDNA or dsDNA. Excess ssDNA completely abolished binding of His_6_MBP-MP fusion to PSTVd RNAs, while dsDNA had no apparent effect (Fig 5E).

Additional positive and negative control experiments were performed to further test the binding properties of the His_6_MBP-MP fusion protein. TMV MP has been reported to bind a 182 nucleotide single stranded fragment of the d24 mutant of the spinach chloroplast *petD* gene [11]. Sense and antisense *petD*-d24 fragments were cloned and *in vitro* transcripts prepared. Sense and antisense fragments were annealed, and double-stranded *petD*-d24 (ds*petD*-d24) RNA was prepared and purified from a native PAGE gel (5%). Then, single-stranded *petD*-d24 (ss*petD*-d24) RNA (sense) and ds*petD*-d24 RNA were used to repeat the competition assay described in Fig 5E. Consistently, binding of His_6_MBP-MP to PSTVd RNAs was abolished by excess ss*petD*-d24 RNA, but not ds*petD*-d24 RNA (Fig 5F). Thus, MP does not selectively bind PSTVd RNA. Binding specificity of His_6_MBP-MP was further tested by incubating 1 ng folded sense *petD*-d24 transcripts with 40 ng His_6_MBP-MP fusion protein alone, or in the presence of 100 ng heterologous ssDNA or dsDNA. His_6_MBP-MP was able to bind the *petD*-d24 transcript, and binding was efficiently competed by ssDNA but not dsDNA (Fig 5G). We next asked whether binding by the fusion protein was due to the His_6_MBP tag, which was prepared by the same procedure used to express the His_6_MBP-MP fusion protein (Fig 5H). Binding was tested by incubating 1 ng WT PSTVd with 40 ng His_6_MBP alone or in the presence of excess ssDNA or dsDNA. No binding was observed under any condition (Fig 5I). Thus, while the His_6_MBP-MP fusion protein has not been tested for its ability to support movement of TMV MP mutants, it is clearly capable of binding single stranded nucleic acids. Given these results, it is unlikely that the inability of TMV MP to mediate phloem entry of 76AU and 156AU is due to reduced binding.

### TMV co-infection, but not TMV MP and CP expression, complements the PSTVd 76AU mutant deficient for phloem entry

It is known that CP is required for systemic infection by TMV [27,28,43]. The PSTVd 76AU and 156AU mutants deficient for phloem entry are capable of spread between other cell types in inoculated leaves, and both are systemically infectious when delivered directly into phloem [22]. Thus, we reasoned that if MP and CP together could support phloem entry in inoculated leaves, systemic infection would be readily observable. To test this, we asked whether transient CP expression could support systemic infection by a TMV CP deletion mutant. First, the CP gene of the TMV-GFP vector was deleted to create TMV-GFP(ΔCP). Then six Nb plants were co-inoculated by agroinfiltration with a pCB301 vector to express TMV-GFP(ΔCP) and a pCAMBIA 1301 vector to express MYC from the 35S promoter. Another six plants were co-inoculated with TMV-GFP(ΔCP) and a pCAMBIA 1301 vector to express TMV CP. Systemic trafficking of TMV-GFP(ΔCP) was monitored by GFP fluorescence under UV light in upper leaves (S4A Fig), and by RT-PCR amplification of TMV MP RNA in extracts obtained from systemic leaves at nine days post agroinfiltration (S4B Fig). In all cases, GFP fluorescence was readily observed on lower leaves at agroinfiltration sites. On systemic leaves, GFP signal was detected only in small regions on leaves of three plants co-inoculated with TMV-GFP(ΔCP) and TMV CP. However, RT-PCR clearly revealed the presence of TMV MP RNA in systemic leaves of all six Nb plants, indicating that transient expression of TMV CP is sufficient to complement trafficking-defective TMV-GFP(ΔCP). In contrast, TMV-GFP(ΔCP) failed to systemically infect any of the six plants on which it was co-inoculated with a construct to express MYC.

To test complementation of PSTVd RNAs, Nb+MP plants were inoculated with *in vitro* transcripts of PSTVd WT, 76AU, and 156AU mutants, followed by agroinfiltration of the same leaves with pCAMBIA 1301 vectors expressing TMV CP or MYC (negative control) at five and 10 dpi. CP expression was confirmed at 15 dpi by RT-PCR (S5 Fig).

**Fig 6:**
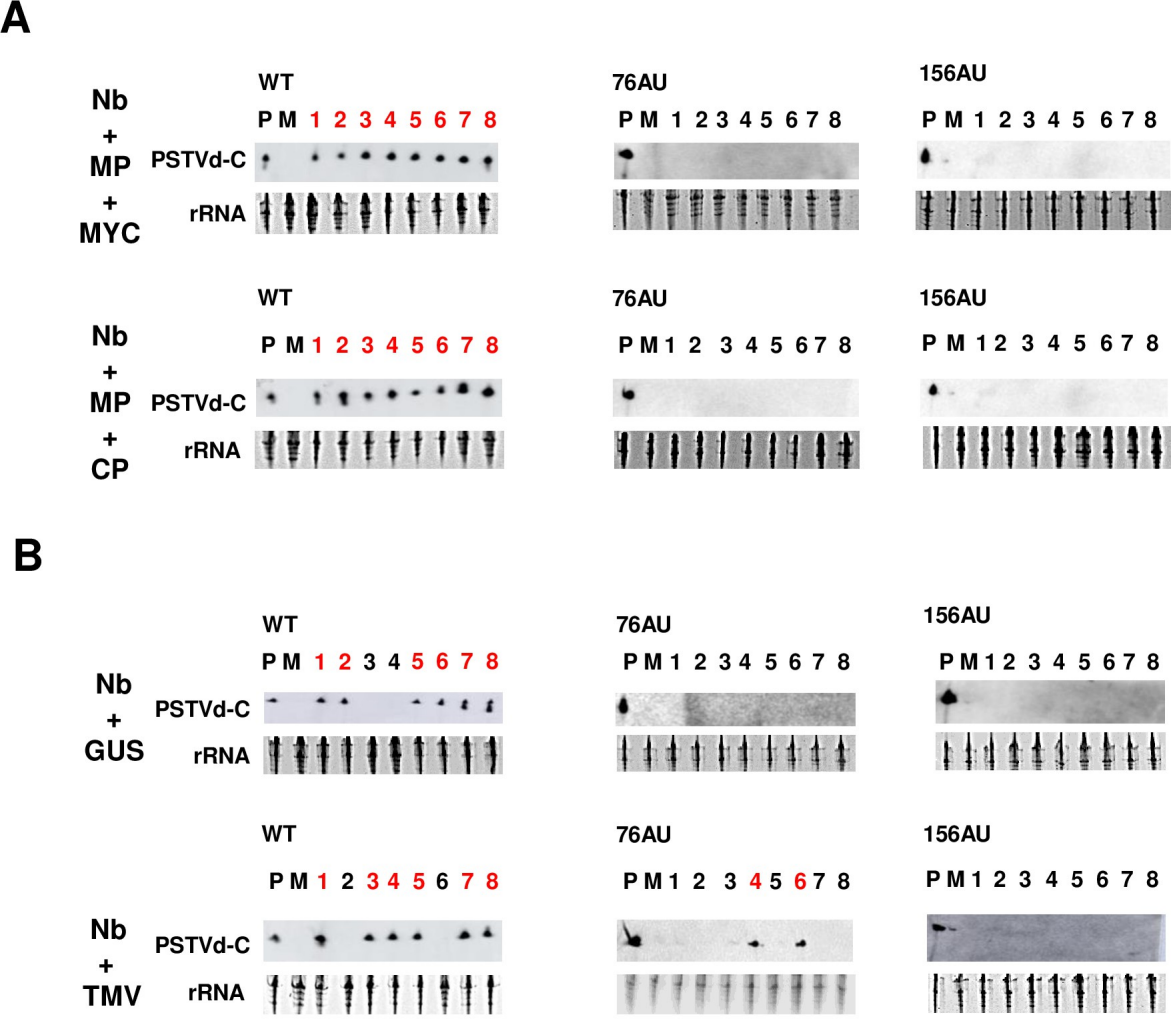
TMV infection, but not TMV MP and CP expression, enables systemic infection of 76AU. (A) *In vitro* transcripts of WT PSTVd and the 76AU and 156AU mutants were rub-inoculated onto leaves of 2-week-old Nb+MP plants, with 8 plants in each group. TMV CP- and MYC-expressing pCAMBIA1301 vectors were delivered into cells of inoculated leaves twice by agroinfiltration at 5 dpi and 10 dpi. Systemic leaves from Nb+MP+CP and Nb+MP+MYC (negative control) plants were collected at 30 dpi. RNA was isolated and RNA blots performed to detect circular PSTVd RNA. (B) *In vitro* transcripts representing WT PSTVd and the 76AU, and 156AU mutants were rub-inoculated onto leaves of 2-week-old Nb plants, with 8 plants in each group. TMV- and GUS-expressing pCB301 vectors were delivered into cells of inoculated leaves by agroinoculation at 3 dpi. Systemic leaves were collected from Nb+TMV and Nb+GUS plants at 20 dpi. RNA was isolated and RNA blots performed to detect circular PSTVd RNA. P, positive control, M, mock. Numbers indicate individual plants, with red indicating positive PSTVd signal. Loading control was rRNA.

In both Nb+MP plants with MYC expression (Nb+MP+MYC) and Nb+MP plants with TMV CP expression (Nb+MP+CP), PSTVd WT, 76AU, and 156AU were detected in inoculated leaves of 8/8 plants at 10 dpi (S6A Fig). Systemic infection was not detected at this time point (S6B Fig). By 20 dpi, WT PSTVd systemically infected 6/8 Nb+MP+MYC plants and 7/8 Nb+MP+CP plants, while 76AU and 156AU failed to systemically infect plants at this time point (S6C Fig). All plants were infected with WT PSTVd after 30 days, while plants inoculated with 76AU and 156AU remained uninfected (Fig 6A). These results suggest that the combination of TMV MP and CP is not sufficient to complement the phloem loading defects of these mutants.

TMV encodes 123 kDa and 183 kDa replicase proteins in addition to MP and CP. To test whether these additional viral factors may also participate in RNA trafficking, and specifically phloem loading, non-transgenic Nb plants rub-inoculated with WT PSTVd, 76AU, or 156AU were agroinoculated with a pCB301 vector expressing TMV or GUS (negative control) at three dpi. Symptoms of TMV infection, including stunting and leaf mosaic, were recorded at 10, 20, and 23 dpi (corresponding to 7, 17, and 20 days after agroinoculation with TMV). PSTVd infection of *N*. *benthamiana* is asymptomatic, but this species is very sensitive to TMV. Accordingly, plants began to exhibit obvious TMV disease symptoms (stunting and leaf mosaic) by 10 dpi and succumbed to lethal necrosis by 23 dpi (S7A Fig). To further confirm TMV infection, CP transcripts were detected in inoculated leaves of these plants by RT-PCR at 10 dpi (S7B Fig).

In both Nb plants with TMV (Nb+TMV) and Nb plants with GUS expression (Nb+GUS), infection by WT PSTVd and the 76AU and 156AU mutants was detected at 10 dpi in inoculated leaves of 8/8 plants (S8A Fig), although no systemic infection was detected by RNA blot (S8B Fig). By 20 dpi, WT PSTVd systemically infected 6/8 Nb+TMV plants and 6/8 Nb+GUS plants (Fig 6B), indicating that TMV co-infection had no general inhibitory effect on replication and spread of the WT viroid. Interestingly, although 156AU failed to systemically infect plants at 20 dpi, 76AU infection was detected in two Nb+TMV plants. Since plant death occurred ~23 dpi, no further experiments were performed.

It is possible that observed systemic spread of 76AU might be a consequence of stress due to severe symptoms of TMV infection at 20 dpi. To detect earlier PSTVd infection, extracts collected from the same plants at 10 dpi, when symptoms were not yet severe (S7A Fig), were re-examined by RT-PCR. As revealed by this more sensitive method, WT PSTVd systemically infected 5/8 Nb+TMV plants and 5/8 Nb+GUS plants at 10 dpi (S9A Fig). The 156AU mutant failed to infect any plants at 10 dpi. Although 76AU failed to systemically infect Nb+GUS plants, it could be detected in two Nb+TMV plants (S9A Fig), the same two in which PSTVd was detected by RNA blot at 20 dpi (Fig 6B). At 20 dpi, wild type PSTVd systemic infection revealed by RT-PCR was completely consistent with RNA blot data (S9B Fig).

Ten progeny clones from the two 76AU infected Nb+TMV plants (five from each plant) were sequenced at 20 dpi. From one plant, all five progeny clones maintained the original 76AU mutation without additional mutations. From the other, two progeny maintained the original mutation without additional mutations, one maintained the mutation but gained another (76AU/G245A), and two reverted to wild type. These results suggest that TMV infection can complement the phloem loading defect of the 76AU mutant, albeit with reduced efficiency, and implicate TMV replicase proteins in phloem loading. However, TMV proteins cannot support entry of the 156AU mutant into the phloem, suggesting that unknown host factors participate in RNA transport at the bundle sheath-phloem boundary. Alternatively, the PSTVd mutants may be unable to effectively interact with TMV proteins other than MP.

## Conclusion

Previous studies showed that expression of TMV MP in tobacco (*N*. *tabacum* cv. Xanthi) increases the SEL of plasmodesmata between mesophyll cells mainly in mature leaves [10,44]. In the same transgenic lines, SEL was also increased between mesophyll and bundle sheath cells, but not between bundle sheath and phloem parenchyma cells, even though MP was observed in plasmodesmata of these cell types [26]. This suggests that MP cannot productively interact with plasmodesmata connecting bundle sheath and phloem parenchyma, which might explain why MP alone is unable to complement PSTVd 76AU and 156AU mutants that are deficient for phloem entry. However, MP accumulation in plasmodesmata of bundle sheath and phloem parenchyma cells, and the ability of MP to increase SEL in the *N*. *benthamiana* plants used in the present study, have not been examined. It is also possible that TMV requires MP and additional viral and cellular proteins, or utilizes a non-MP mediated mechanism, to load into the phloem. Consequently, we tested and found that co-infection with TMV, but not the combination of MP and CP expression, could complement the 76AU mutant, suggesting a role for TMV replicase proteins in phloem loading. In contrast, the 156AU mutant was not complemented by either condition, implying that TMV proteins alone are insufficient in this case and implicating host factors in phloem loading. Alternatively, PSTVd RNAs may not effectively interact with TMV proteins other than MP. Nevertheless, TMV MP alone is sufficient to mediate transport of GG mutant PSTVd RNA, and by extension TMV RNA, between upper epidermis and palisade mesophyll cells, validating this approach for testing the RNA trafficking functions of MPs.

It should be noted that transgenic Nb plants expressing TMV MP, as well as plants expressing the MP of *Red clover necrotic mottle virus* (RCNMV), have previously been shown to support cell-to-cell and systemic spread of heterologous viral RNAs, including that of insect-infecting *Flock house virus* [45]. On the other hand, our results indicate that by itself, TMV MP cannot support systemic movement. Thus, an important advantage of PSTVd, illustrated here, is the availability of RNA mutants defective for directional movement through specific plasmodesmal gates, allowing analysis of MP trafficking function in fine detail. It is also interesting to note that proteins of different viruses can have different activities. For example, both the MP and CP of *Brome mosaic virus* (BMV) are required for cell-to-cell spread and long distance movement [46].

In summary, this study reports a novel method to analyze the function of movement proteins. Utilizing PSTVd mutants defective for movement between different cell types, the ability of TMV MP to mediate RNA trafficking between upper epidermis and palisade mesophyll, and between bundle sheath and phloem, was tested. We showed that TMV MP enabled mesophyll entry of the GG mutant which is defective for this process, indicating TMV MP is capable by itself of mediating RNA trafficking from epidermal cells to mesophyll cells. In contrast, TMV MP, and the combination of MP and CP expression, failed to support systemic infection by the 76AU and 156AU mutants defective for phloem entry. However, TMV infection complemented 76AU but not 156AU, suggesting the involvement of viral replicase proteins and unidentified cellular factors in phloem entry in addition to MP. These observations confirm that different gating mechanisms exist between different cell types and highlight the complexity of phloem loading compared to other types of cell-to-cell movement, suggestive of a role as gatekeeper to the vascular system.

## Methods

### Plants and inoculation

Nb+MP plants [35], a gift from Dr. James Culver, were grown in The Ohio State University Biotechnology Support Facility along with wild type Nb plants. Plasmid pRZ6-2 containing WT PSTVd was a gift from Dr. Robert Owens. Plasmids carrying the cDNAs of WT PSTVd and three mutants (GG, 76AU, and 156AU) were linearized with Hind III (Promega), followed by *in vitro* transcription using the MEGAscript T7 Transcription Kit (ThermoFisher Scientific). PCR products containing the T7 promoter region and the 182 bp sense and antisense spinach *pet*D-d24 sequences were also subjected to *in vitro* transcription using MEGAscript T7 Transcription Kit to prepare *pet*D-d24 RNA. PSTVd and *pet*D-d24 riboprobes labeled with digoxigenin (DIG) were prepared using MAXIscript T3 Transcription Kit (ThermoFisher Scientific) and MAXIscript T7 Transcription Kit (ThermoFisher Scientific), respectively. Templates were plasmid pInter(-) linearized with SpeI and a PCR product containing T7 promoter and *pet*D-d24 antisense sequence, respectively. After purification using MEGAclear Transcription Clean-Up Kit (ThermoFisher Scientific), RNA transcripts (300 ng) were rub-inoculated onto the upper surface of first two true leaves of 2-week-old Nb and Nb+MP plants that were previously dusted with carborundum powder [21].

#### RNA isolation and blotting

Plant RNA samples were prepared using Trizol reagent (Invitrogen) according to the manufacturer’s instructions. Prior to subsequent experiments, RNA samples were incubated with DNase I (Invitrogen) at 37°C for 2 h to remove genomic DNA. RNA blotting was performed essentially as described [47], except digoxigenin (DIG)-labeled probe instead of [α-^32^P]-UTP-labeled probe was used in the present study. To prepare (DIG)-labeled PSTVd riboprobes, pInter(-) plasmid was linearized with SpeI (New England Biolabs) [48]. This was followed by *in vitro* transcription using the MAXIscript T7 Transcription Kit (ThermoFisher Scientific). Signal was captured using myECL Imager (ThermoFisher Scientific).

#### RT-PCR

Reverse transcription was performed with Superscript III Reverse Transcriptase (ThermoFisher Scientific) using RNA samples from indicated plants as template. Reverse primers MPdetect-R (5’-TGGGCCCTCCGTCTCTCACG-3’), CPdetect-R (5’-CAAACCAGAAGAGCTCTCGAAAG-3’) and actindetect-R (5’-CAGGATACGGGGAGCTAATGC-3’) were used to detect MP, CP and actin expression, respectively. To clone the MP, CP, and MBP open reading frames (ORFs), reverse primers MPclone-R (5’-TTAAAACGAATCCGATTCGG-3’), CPclone-R (5’-GAGGAGAAGAGCCGTCGTCAAGTTGCAGGACCAGAG-3’), and MBPclone-R (5’-CAGTGGTGGTGGTGGTGGTGCTCGAGTTAAGTCTGCGCGTCTTTCAGGGCT-3’) were employed, respectively. To prepare DNA fragments composed of T7 promoter region and 182 bp *pet*D-d24 in sense and antisense orientation, reverse primers *pet*D-d24-senseR (5’-GTATCTAGGGAATAGTCACT-3’) and *pet*D-d24-antisenseR (5’-GTTAATAAATTCCAAAATCCAT-3’) were used. Reverse primer PSTVd-R (5’- GCTTCAGTTGTTTCCACCGG-3’) was used to clone PSTVd progeny. PCR reactions were performed with MPdetect-R and MPdetect-F (5’-AGCCGGTTTGGTCGTCACGG-3’), CPdetect-R and CPdetect-F (5’-CTCCATCTCAGTTCGTGTTCTTG-3’), actindetect-R and actindetect-F (5’-AACTGATGAAGATACTCACAG-3’), MPclone-R and MPclone-F (5’-ATGGCTCTAGTTGTTAAAGG-3’), CPclone-R and CPclone-F (5’-CGACGACAAGACCGTCACCATGTCTTACAGTATCACTACTC-3’), MBPclone-R and MBPclone-F (5’-CAGTGGTGGTGGTGGTGGTGCTCGAGTTAAGTCTGCGCGTCTTTCAGGGCT-3’), *pet*D-d24-senseR and *pet*D-d24-senseF (5’-TAATACGACTCACTATAGTTAATAAATTCCAAAATCC-3’), *pet*D-d24-antisenseR and *pet*D-d24-antisenseF (5’-TAATACGACTCACTATAGAGTGACTATTCCCTAGATACAC-3’), or PSTVd-R and PSTVd-F (5’-GCTGTCGCTTCGGCTACTAC-3’). To prepare TMV-GFP (TE1), site-directed mutagenesis was performed using TE1-F (5’AAAGGAAAAGTGATATCAATGAGTTT-3’) and TE1-R (5’-AAACTCATTGATATCACTTTTCCTTT-3’) primers. To prepare TMV-GFP (ΔCP) mutant, PCR products were prepared using ΔCP-F (5’-AAGTTCATTTCATTTGGAGAGGGTATTTTTACAACAATTACCAACAACAACAAAC-3’), ΔCP-R1 and (5’TGGGCCCCTACCGGGGGTAACGGGGGGATTCGAACCCCTCGCTTTATTACGTG -3’) PCR was carried out using Phusion High-Fidelity DNA Polymerase (New England Biolabs). PCR products were subjected to 1.5% agarose gel electrophoresis, and images were taken after ethidium bromide staining.

#### PSTVd progeny sequencing

Full-length progeny sequences were amplified from greater than full-length reverse transcription products as described [22], using forward primer PSTVd1F (5’-CGGAACTAAACTCGTGGTTCCT-3’) and reverse primer PSTVd1S (5’-AGGAACCAACTGCGGTTCCA-3’). RT-PCR amplified progeny products were purified through agarose gel using the Zymoclean Gel DNA Recovery Kit (Zymo Research). Purified products were cloned into PCR2.1 using a TA Cloning Kit (Invitrogen). DNA sequencing was performed at the Genomics Shared Resource of The Ohio State University.

#### Vector construction

The entire TMV MP ORF was amplified from NB+MP plants and purified following agarose gel electrophoresis using the Zymoclean Gel DNA Recovery Kit. The ORF was then cloned into the pCR8 TOPO gateway vector using the pCR8/GW/TOPO TA Cloning Kit (ThermoFisher Scientific). Through an LR reaction using Gateway LR Clonase Enzyme mix (ThermoFisher Scientific), the MP ORF was cloned into the pDEST-HisMBP vector for expression as a His6 maltose binding protein fusion (His_6_MBP-MP) [49]. Constructs were sequenced to ensure correct sequence and orientation. To construct the His_6_MBP expression vector, the entire MBP cDNA was amplified from pDEST-HisMBP vector. After gel purification, MBP cDNA was cloned into pET-28a expression vector carrying N-terminal His tag through homologous recombination. Through the same method, pCB301 vector carrying full-length TMV, kindly provided by Dr. Jiejun Peng at Ningbo University, was used as a template to clone TMV CP full length cDNA. CP cDNA was cloned into the pCAMBIA1301 vector to establish CP-expressing vector using T4 DNA Polymerase (ThermoFisher Scientific)-mediated ligation. MYC-expressing pCAMBIA1301 vector, TMV-GFP-expressing pCB301 vector and GUS-expressing pCB301 vector were kindly provided by Dr. Kelei Han at Ningbo University. The TMV-GFP(TE1) mutant was constructed by site-directed mutagenesis. The TMV-GFP(ΔCP) mutant was prepared by homologous recombination.

#### Agroinoculation

TMV CP-, and MYC-expressing pCAMBIA1301 vectors, as well as TMV-GFP(TE1), TMV-GFP(ΔCP), TMV- and GUS-expressing pCB301 vectors, were transfected into *Agrobacterium tumefaciens*. Transfected agrobacteria were cultivated in LB medium containing 25 μg/ml kanamycin and 100 μg/ml rifampicin at 28°C overnight. The next day, agrobacteria were collected and resuspended in MMA solution (10 mM MgCl_2_, 100 μM acetosyringone, 10 mM MES pH 5.6). To perform TMV infection at 3 days post PSTVd inoculation (dpi), OD values were adjusted to 0.1. To express TMV CP at 5 and 10 dpi, OD values were adjusted to 0.5.

#### Protein purification

The pDEST-HisMBP vector carrying TMV MP and pET-28a vector carrying MBP were transfected into *E*. *coli* strain BL21 (EMD Millipore). Bacteria were cultivated at 37°C until a A600 value close to 0.6 was reached. IPTG was added to 0.1 mM and, following incubation for a further 4 h, cultures were centrifuged at 5000 g for 15 min at 4°C. To extract protein, cell pellets were resuspended in native binding buffer (50 mM NaH_2_PO_4_, 0.5 M NaCl, pH 8.0, 0.1% (v/v) Triton X-100). Lysozyme (1 mg/ml) was then added to the cell suspension. Cell lysis was performed on ice for 30 min. After sonication, cell lysates were centrifuged at 5000 g for 15 min at 4°C to collect supernatant, which was incubated with Ni-NTA agarose (Qiagen) to adsorb His tagged fusion protein. Ni-NTA agarose and bound protein were collected by centrifugation. After washing the pellet with native washing buffer (50 mM NaH_2_PO_4_, pH 8.0, 0.5 M NaCl, 20 mM imidazole), fusion protein was eluted using native elution buffer (50 mM NaH_2_PO_4_, pH 8.0, 0.5 M NaCl, 0.25 M imidazole). Gel filtration was performed with Sephadex G-25 beads (Sigma-Aldrich) prior to subsequent assays. Proteins were visualized by Coomassie blue staining of a 10% SDS-polyacrylamide (37.5:1) gel.

#### RNA folding and EMSA

*In vitro* transcripts of WT PSTVd and the GG, 76AU and 156AU mutants, as well as spinach chloroplast *pet*D-d24, were prepared and folded as previously described [22]. RNA folding was evaluated by 6% native polyacrylamide gel (29:1) electrophoresis. Electrophoresis was performed in a cold room (4°C) at 100V for 2 h. Folded RNA samples (1 ng) were incubated with increasing amounts of His_6_MBP-MP fusion protein (0, 10, 20, 40, 80, 160, and 320 ng) in 10 μl binding buffer (20 mM Tris-HCl, pH 7.5, 35 mM KCl, 3.5 mM MgCl_2_ and 5U RNaseOUT) (ThermoFischer Scientific) for 30 min at 4°C. Binding products were subjected to 6% native polyacrylamide gel (29:1) electrophoresis at 4°C for 3h at 120V. RNA blots were performed to analyze binding.

In competition assays, PSTVd or *pet*D-d24 transcripts (1 ng) were incubated with 40 ng His_6_MBP-MP fusion protein or 40 ng His_6_MBP protein alone, or in the presence of 100 ng ssDNA, or dsDNA. To prepare dsDNA samples, a mixture of pCR8 TOPO TA vector, pGEM-T easy vector, and pDEST-HisMBP vector (1:1:1 ratio, w/w) was first digested with RNase A overnight to remove all potential RNA contamination, followed by three rounds of phenol-chloroform extraction to remove RNase A. To prepare ssDNA samples, the three plasmids were linearized using EcoRI, SpeI, and EcoRI, respectively. Plasmid mixtures were incubated at 98°C for 10 min, followed by freezing in liquid nitrogen prior to use. To prepare ds*pet*D-d24 RNA, sense and antisense *pet*D-d24 RNAs were mixed (1:1 ratio, w/w). The mixture was denatured at 98°C for 10 min, followed by slow cooling to room temperature. The resulting ds*pet*D-d24 RNA was purified twice through 5% native PAGE gel electrophoresis to completely remove ssRNA.

#### *In situ* hybridization

Preparation of inoculated leaf transverse paraffin sections and *in situ* hybridization was performed as described [21]. Two inoculated leaves from each of three GG, 76AU, or 156AU mutant infected Nb and Nb+MP plants were used. Two sections were prepared from each leaf, and four visual fields were examined per section.

## Supporting information

S1 Fig Confirmation of MP expression in transgenic *N*. *benthamiana*. The presence of TMV MP transcripts in extracts obtained from nine randomly selected Nb+MP plants was confirmed by RT-PCR using MP-specific primers. Actin mRNA served as an internal control. M, non-transgenic Nb plant.
(TIF)

S2 Fig Transgenic MP expression enables systemic trafficking of the TMV-GFP(TE1) mutant. Six Nb and Nb+MP plants were co-inoculated on two lower leaves by agroinfiltration with pCB301 vectors to express the trafficking-defective TMV-GFP(TE1) mutant and GUS. As a positive control, another six Nb plants were co-inoculated with pCB301 expressing TMV-GFP(TE1) and a second pCB301 vector expressing TMV. (A) GFP fluorescence was monitored under UV light nine days post-inoculation. Uniform dark red color in the absence of GFP is due to chlorophyll autofluorescence. As indicated by GFP signal in upper leaves, systemic spread of TMV-GFP(TE1) was observed in Nb+MP plants, and in Nb plants when co-inoculated with TMV. In Nb plants co-inoculated with TMV-GFP(TE1) and GUS, GFP fluorescence was confined to agroinfiltration sites on lower leaves. (B) MP expression was monitored by RT-PCR, with actin as an endogenous control. P, positive control. M, mock. Extracts were obtained from upper, systemic leaves at 9 days post-inoculation. In Nb+MP plants, expression can be attributed to the 35S-MP transgene and the TMV-GFP(TE1) mutant, whereas MP is expressed from virus in Nb plants co-inoculated with TMV and TMV-GFP(TE1). However, presence of the TE1 mutant sequence was confirmed by progeny sequencing (six plants in each group).
(TIF)

S3 Fig Infectivity of WT PSTVd and mutants GG, 76AU, and 156AU at 10 dpi. (A) Rub-inoculated leaves of the same Nb and Nb+MP plants noted in Fig 4 were collected at 10 dpi and RNA samples analyzed for PSTVd accumulation by RNA blot. (B) RNA blot signals presented in (A) were quantified using Quantity One software. For each treatment the Nb+MP group was normalized to the Nb group, which was set to 1. Mean +/- SD values are shown for the eight plants in each group. Asterisk indicates significant difference (p < 0.05) by Student’s *t* test. (C) Systemic infection assay at 10 dpi. Upper leaves from the same plants noted in Fig 4 were collected at 10 dpi and analyzed for PSTVd accumulation. Numbers indicate the 8 plants included in each group. None were positive for PSTVd at this time point. P, positive control. M, mock. PSTVd-C, circular form of PSTVd. Loading control was ribosomal RNA.
(TIF)

S4 Fig Transient CP expression enables systemic trafficking of the TMV-GFP(ΔCP) mutant. Six Nb plants were co-agroinfiltrated with a pCB301 vector expressing trafficking-defective TMV-GFP(ΔCP) mutant and a pCAMBIA1301 vector expressing MYC. Another six Nb plants were co-agroinfiltrated with a pCAMBIA1301 vector expressing TMV CP and a pCB301 vector expressing trafficking-defective TMV-GFP(ΔCP) mutant. (A) GFP signal indicating systemic trafficking of TMV-GFP(ΔCP) was detected at nine days post agroinfiltration. (B) Systemic trafficking of TMV-GFP(ΔCP) was also confirmed by RT-PCR amplification of TMV MP with actin as an endogenous control. P, positive control. M, mock.
(TIF)

S5 Fig Confirmation of TMV CP expression in Nb+MP plants following Agrobacterium-mediated delivery of a CP expression construct. Nb+MP plants were first inoculated with WT PSTVd or the 76AU and 156AU mutants, followed by agroinoculation of the same leaves with pCAMBIA vectors expressing TMV CP (Nb+MP+CP) or MYC (Nb+CP+MYC, negative control) at 5 and 10 dpi. TMV CP expression was confirmed at 15 dpi by RT-PCR with actin as an endogenous control. P, positive control. M, mock.
(TIF)

S6 Fig Infectivity of WT PSTVd and 76AU and 156AU mutants in Nb+MP plants with TMV CP expression. (A) Local infection assay at 10 dpi. Rub-inoculated leaves of Nb+MP plants also expressing TMV CP (Nb+MP+C) or MYC (Nb+CP+MYC, negative control) noted in Fig 6A were collected, and RNA samples analyzed for PSTVd accumulation by RNA blot. (B) Systemic infection assay at 10 dpi. Upper leaves from the same plants were collected and analyzed for PSTVd accumulation. (C) Systemic infection assay at 20 dpi. Numbers indicate the 8 plants included in each group and those in red indicate plants with positive PSTVd signal. P, positive control. M, mock. PSTVd-C, circular form of PSTVd. Loading control was ribosomal RNA.
(TIF)

S7 Fig Confirmation of TMV infection in Nb plants. Nb plants rub-inoculated with WT PSTVd, 76AU, or 156AU were agroinfiltrated at 3 dpi with a pCB301 vector expressing TMV or GUS (negative control) at 3 dpi. (A) Nb+TMV and Nb+GUS (negative control) plants (eight per treatment, noted in Fig 6B) were monitored for symptoms of TMV at 10, 20 and 23 dpi (7, 17 and 20 days after agroinfiltration). TMV infected plants experienced lethal necrosis by 23 days. (B) TMV infection in inoculated leaves of plants presented in Fig 6B was confirmed by RT-PCR amplification of CP sequence at 10 dpi with actin as an endogenous control. P, positive control. M, mock.
(TIF)

S8 Fig Infectivity of PSTVd WT, 76AU, and 156AU in Nb plants with TMV infection at 10 dpi; RNA blot analysis. (A) Local infection assay at 10 dpi. Rub-inoculated leaves of the same Nb+TMV and Nb+GUS plants noted in Fig 6B were collected and RNA samples analyzed for PSTVd accumulation by RNA blot. (B) Systemic infection assay at 10 dpi. Upper leaves from the same plants were collected at 10 dpi and analyzed for PSTVd accumulation. Numbers indicate the 8 plants included in each group and those in red indicate plants with positive PSTVd signal. P, positive control. M, mock. PSTVd-C, circular form of PSTVd. Loading control was ribosomal RNA.
(TIF)

S9 Fig Systemic infection of PSTVd WT, 76AU, and 156AU in Nb plants with TMV infection at 10 and 20 dpi; RT-PCR analysis. RNA samples isolated from rub-inoculated leaves of the same Nb+TMV and Nb+GUS plants noted in Fig 6B were subjected to RT-PCR to detect PSTVd. Systemic infection by PSTVd was detected at 10 (A) and 20 (B) dpi with actin as an endogenous control. PSTVd-C, circular form of PSTVd.
(TIF)
