# Supplementary figures and images for: Tobacco mosaic virus movement protein complements a Potato spindle tuber viroid RNA mutant impaired for mesophyll entry but not mutants unable to enter the phloem
Source: PLoS Pathog. 2022 Dec 27;18(12):e1011062. doi: 10.1371/journal.ppat.1011062 (PMC9829174; doi:10.1371/journal.ppat.1011062)

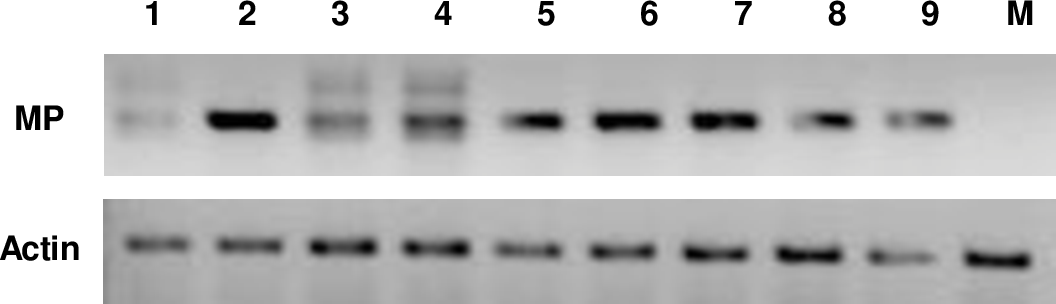

Supplement: S1 Fig — The presence of TMV MP transcripts in extracts obtained from nine randomly selected Nb+MP plants was confirmed by RT-PCR using MP-specific primers. Actin mRNA served as an internal control. M, non-transgenic Nb plant. (TIF) [file ppat.1011062.s001.tif]

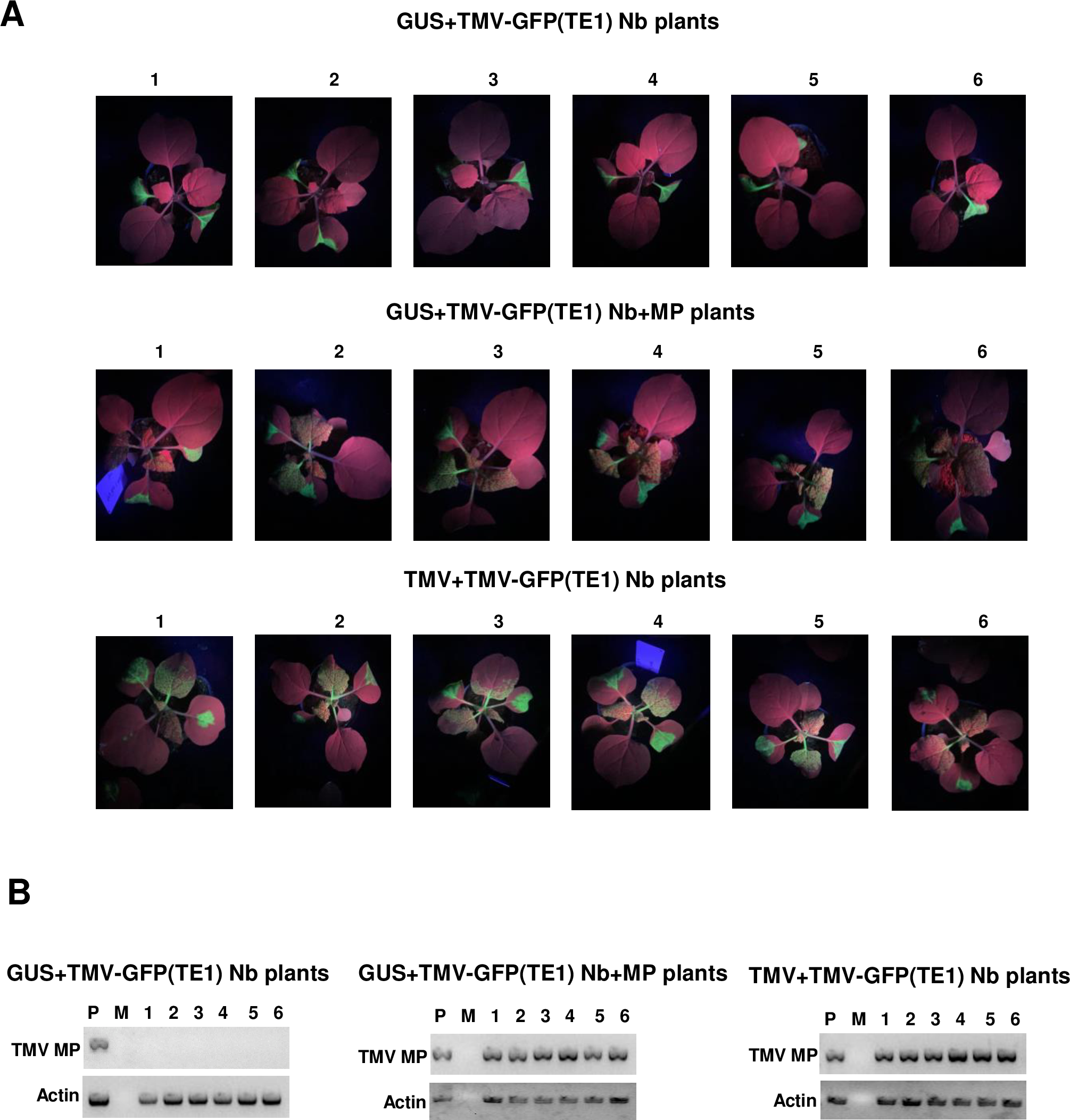

Supplement: S2 Fig — Six Nb and Nb+MP plants were co-inoculated on two lower leaves by agroinfiltration with pCB301 vectors to express the trafficking-defective TMV-GFP(TE1) mutant and GUS. As a positive control, another six Nb plants were co-inoculated with pCB301 expressing TMV-GFP(TE1) and a second pCB301 vector expressing TMV. (A) GFP fluorescence was monitored under UV light nine days post-inoculation. Uniform dark red color in the absence of GFP is due to chlorophyll autofluorescence. As indicated by GFP signal in upper leaves, systemic spread of TMV-GFP(TE1) was observed in Nb+MP plants, and in Nb plants when co-inoculated with TMV. In Nb plants co-inoculated with TMV-GFP(TE1) and GUS, GFP fluorescence was confined to agroinfiltration sites on lower leaves. (B) MP expression was monitored by RT-PCR, with actin as an endogenous control. P, positive control. M, mock. Extracts were obtained from upper, systemic leaves at 9 days post-inoculation. In Nb+MP plants, expression can be attributed to the 35S-MP transgene and the TMV-GFP(TE1) mutant, whereas MP is expressed from virus in Nb plants co-inoculated with TMV and TMV-GFP(TE1). However, presence of the TE1 mutant sequence was confirmed by progeny sequencing (six plants in each group). (TIF) [file ppat.1011062.s002.tif]

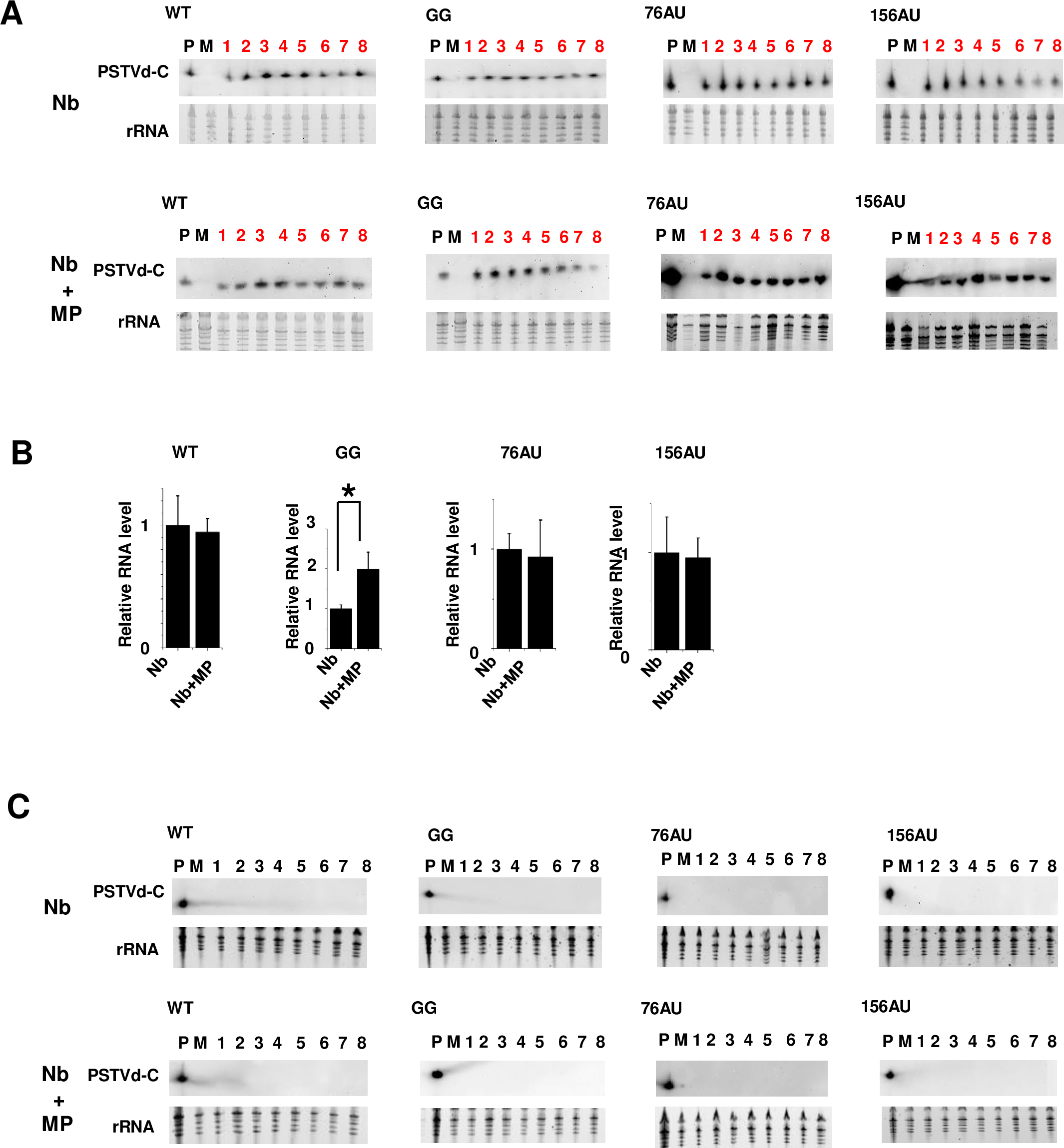

Supplement: S3 Fig — (A) Rub-inoculated leaves of the same Nb and Nb+MP plants noted in Fig 4 were collected at 10 dpi and RNA samples analyzed for PSTVd accumulation by RNA blot. (B) RNA blot signals presented in (A) were quantified using Quantity One software. For each treatment the Nb+MP group was normalized to the Nb group, which was set to 1. Mean +/- SD values are shown for the eight plants in each group. Asterisk indicates significant difference (p < 0.05) by Student’s t test. (C) Systemic infection assay at 10 dpi. Upper leaves from the same plants noted in Fig 4 were collected at 10 dpi and analyzed for PSTVd accumulation. Numbers indicate the 8 plants included in each group. None were positive for PSTVd at this time point. P, positive control. M, mock. PSTVd-C, circular form of PSTVd. Loading control was ribosomal RNA. (TIF) [file ppat.1011062.s003.tif]

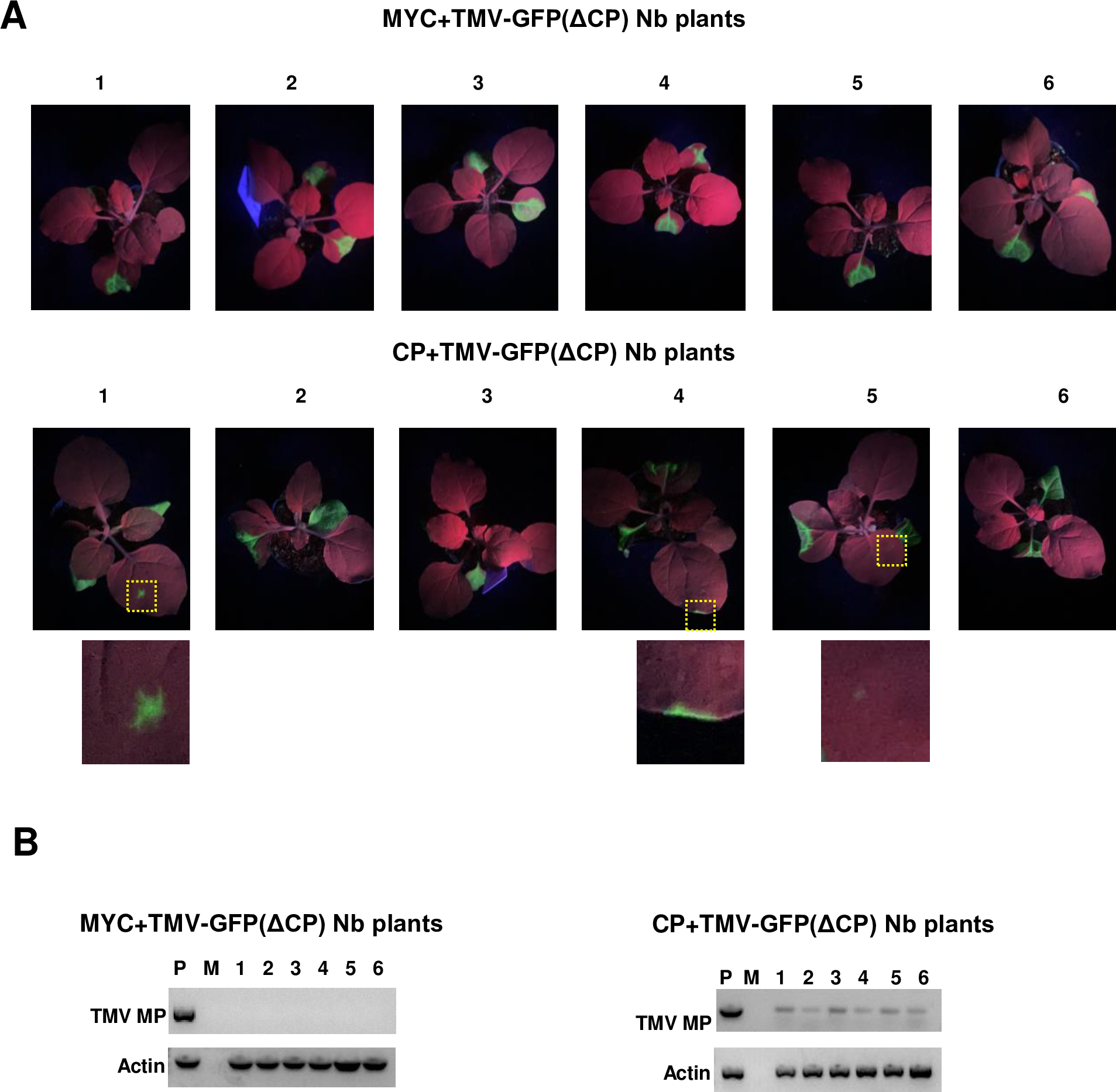

Supplement: S4 Fig — Six Nb plants were co-agroinfiltrated with a pCB301 vector expressing trafficking-defective TMV-GFP(ΔCP) mutant and a pCAMBIA1301 vector expressing MYC. Another six Nb plants were co-agroinfiltrated with a pCAMBIA1301 vector expressing TMV CP and a pCB301 vector expressing trafficking-defective TMV-GFP(ΔCP) mutant. (A) GFP signal indicating systemic trafficking of TMV-GFP(ΔCP) was detected at nine days post agroinfiltration. (B) Systemic trafficking of TMV-GFP(ΔCP) was also confirmed by RT-PCR amplification of TMV MP with actin as an endogenous control. P, positive control. M, mock. (TIF) [file ppat.1011062.s004.tif]

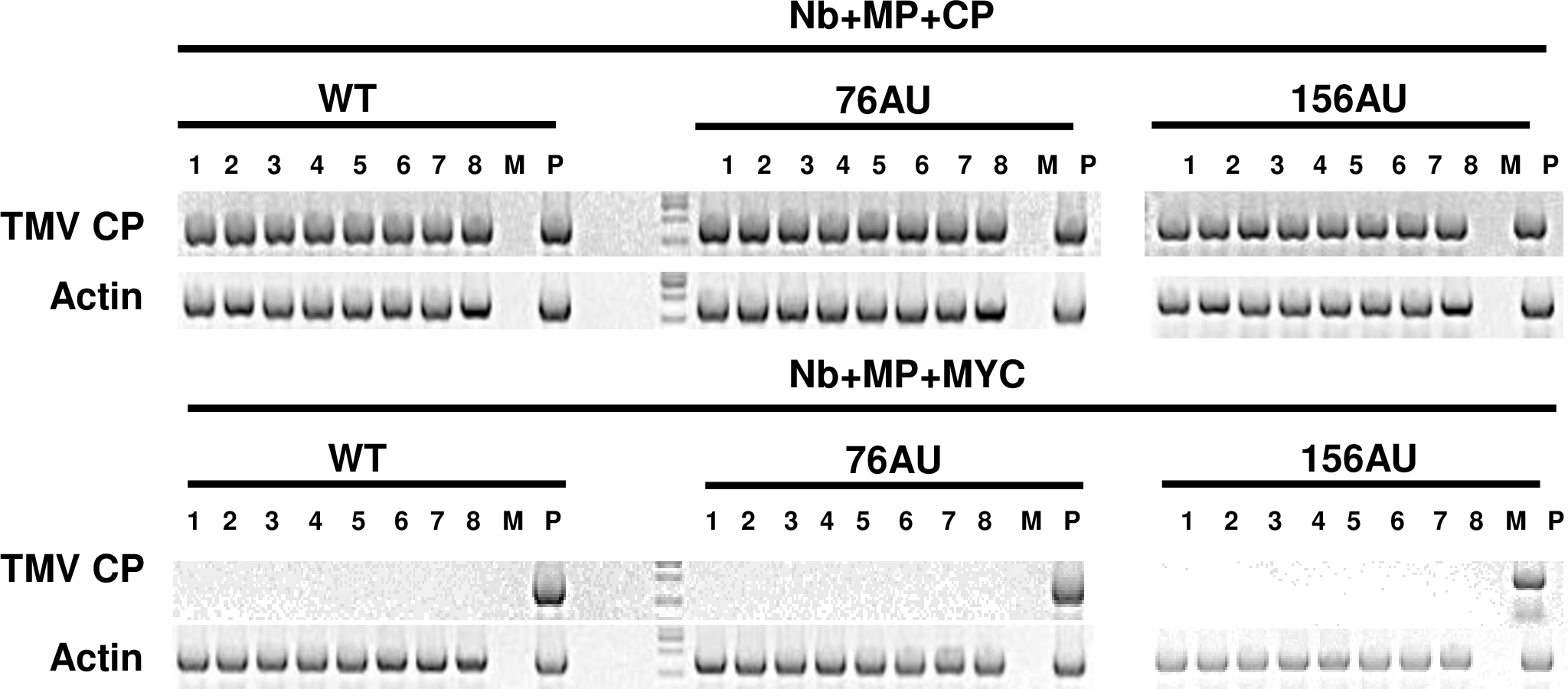

Supplement: S5 Fig — Nb+MP plants were first inoculated with WT PSTVd or the 76AU and 156AU mutants, followed by agroinoculation of the same leaves with pCAMBIA vectors expressing TMV CP (Nb+MP+CP) or MYC (Nb+CP+MYC, negative control) at 5 and 10 dpi. TMV CP expression was confirmed at 15 dpi by RT-PCR with actin as an endogenous control. P, positive control. M, mock. (TIF) [file ppat.1011062.s005.tif]

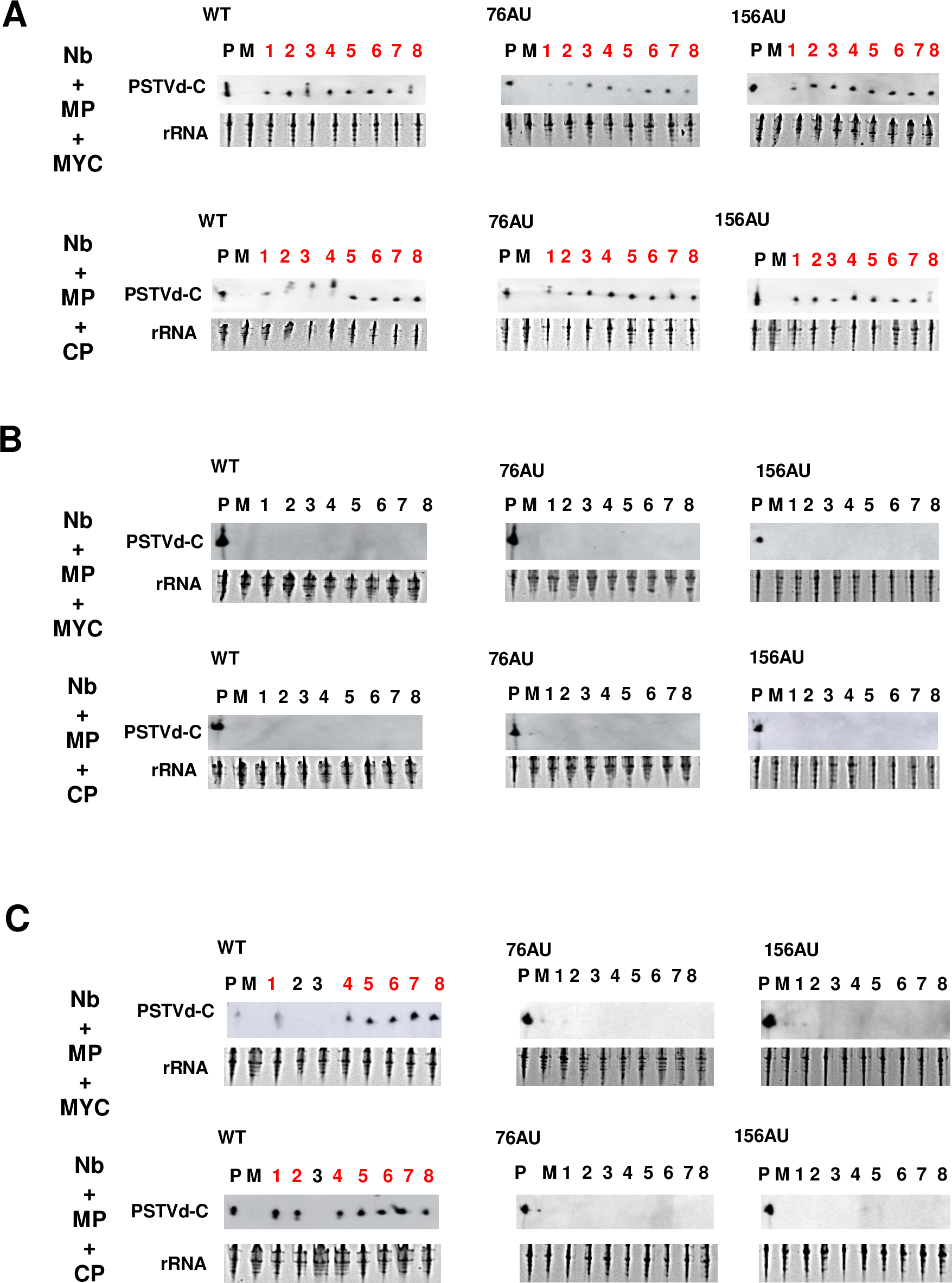

Supplement: S6 Fig — (A) Local infection assay at 10 dpi. Rub-inoculated leaves of Nb+MP plants also expressing TMV CP (Nb+MP+C) or MYC (Nb+CP+MYC, negative control) noted in Fig 6A were collected, and RNA samples analyzed for PSTVd accumulation by RNA blot. (B) Systemic infection assay at 10 dpi. Upper leaves from the same plants were collected and analyzed for PSTVd accumulation. (C) Systemic infection assay at 20 dpi. Numbers indicate the 8 plants included in each group and those in red indicate plants with positive PSTVd signal. P, positive control. M, mock. PSTVd-C, circular form of PSTVd. Loading control was ribosomal RNA. (TIF) [file ppat.1011062.s006.tif]

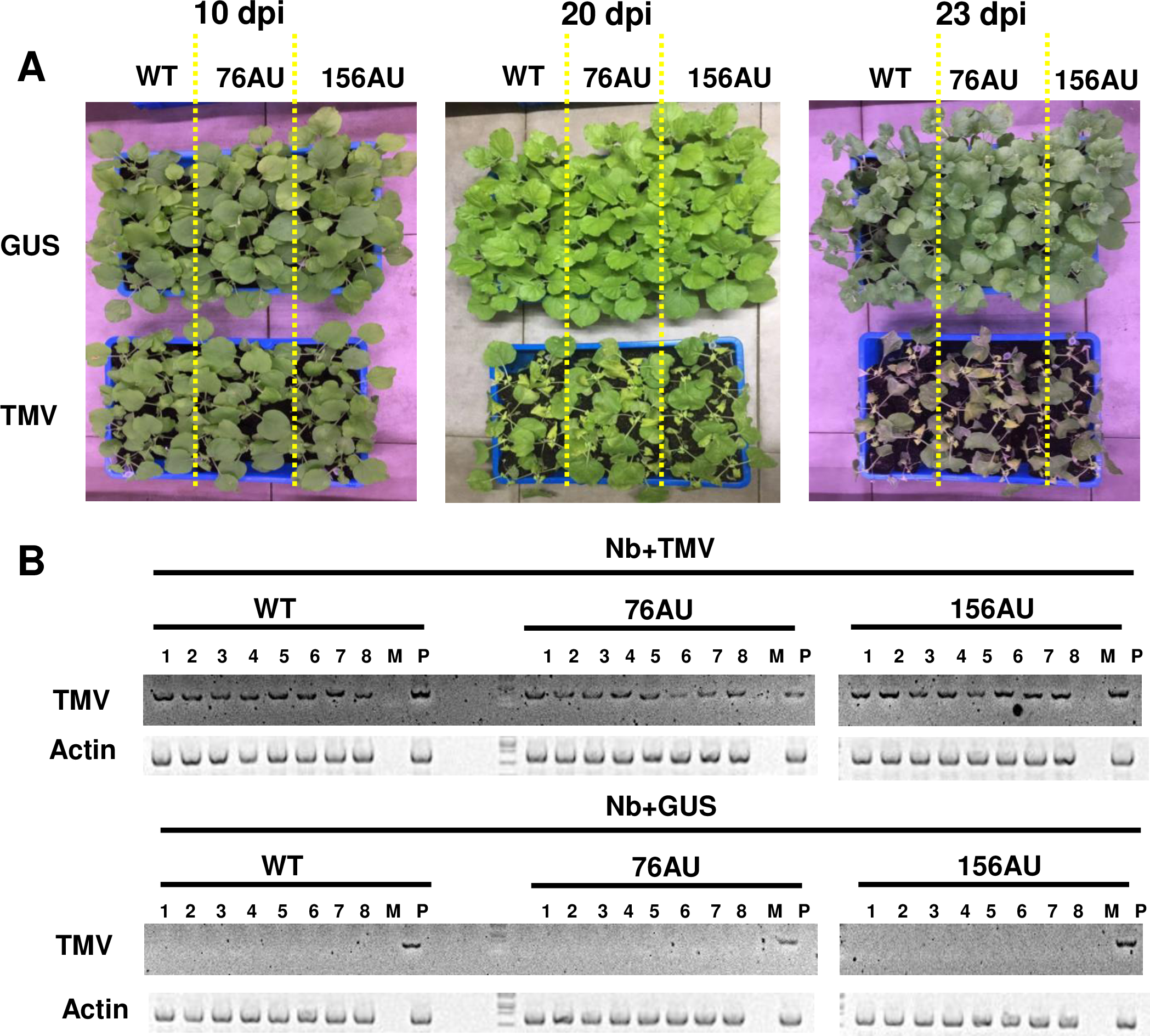

Supplement: S7 Fig — Nb plants rub-inoculated with WT PSTVd, 76AU, or 156AU were agroinfiltrated at 3 dpi with a pCB301 vector expressing TMV or GUS (negative control) at 3 dpi. (A) Nb+TMV and Nb+GUS (negative control) plants (eight per treatment, noted in Fig 6B) were monitored for symptoms of TMV at 10, 20 and 23 dpi (7, 17 and 20 days after agroinfiltration). TMV infected plants experienced lethal necrosis by 23 days. (B) TMV infection in inoculated leaves of plants presented in Fig 6B was confirmed by RT-PCR amplification of CP sequence at 10 dpi with actin as an endogenous control. P, positive control. M, mock. (TIF) [file ppat.1011062.s007.tif]

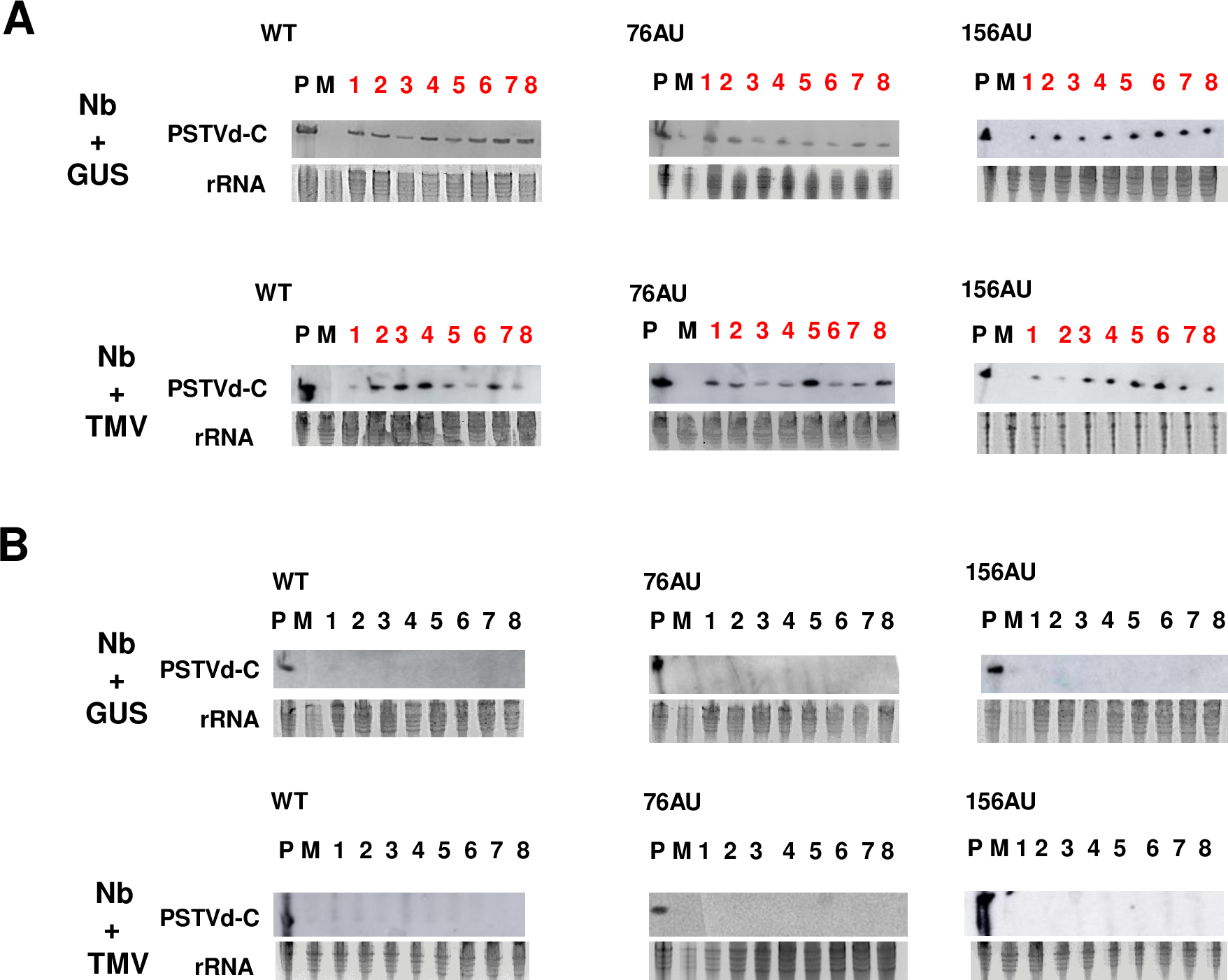

Supplement: S8 Fig — (A) Local infection assay at 10 dpi. Rub-inoculated leaves of the same Nb+TMV and Nb+GUS plants noted in Fig 6B were collected and RNA samples analyzed for PSTVd accumulation by RNA blot. (B) Systemic infection assay at 10 dpi. Upper leaves from the same plants were collected at 10 dpi and analyzed for PSTVd accumulation. Numbers indicate the 8 plants included in each group and those in red indicate plants with positive PSTVd signal. P, positive control. M, mock. PSTVd-C, circular form of PSTVd. Loading control was ribosomal RNA. (TIF) [file ppat.1011062.s008.tif]

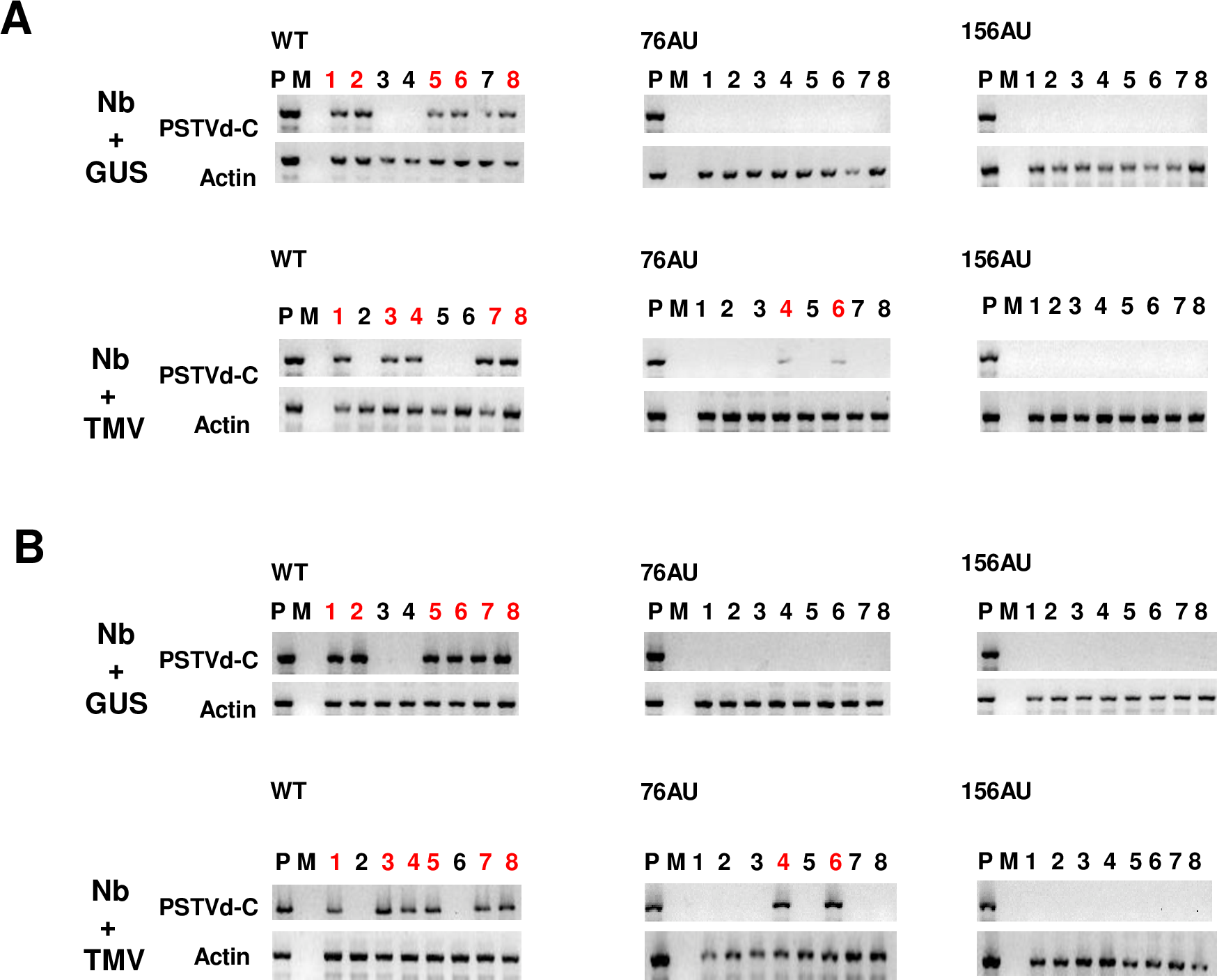

Supplement: S9 Fig — RNA samples isolated from rub-inoculated leaves of the same Nb+TMV and Nb+GUS plants noted in Fig 6B were subjected to RT-PCR to detect PSTVd. Systemic infection by PSTVd was detected at 10 (A) and 20 (B) dpi with actin as an endogenous control. PSTVd-C, circular form of PSTVd. (TIF) [file ppat.1011062.s009.tif]
